# Effect of methylene blue on the genomic response to reperfusion injury induced by cardiac arrest and cardiopulmonary resuscitation in porcine brain

**DOI:** 10.1186/1755-8794-3-27

**Published:** 2010-07-01

**Authors:** Cécile Martijn, Lars Wiklund

**Affiliations:** 1 Department of Surgical Sciences/Anaesthesiology and Intensive Care Medicine, Uppsala University Hospital, SE-751 85 Uppsala, Sweden

## Abstract

**Background:**

Cerebral ischemia/reperfusion injury is a common secondary effect of cardiac arrest which is largely responsible for postresuscitative mortality. Therefore development of therapies which restore and protect the brain function after cardiac arrest is essential. Methylene blue (MB) has been experimentally proven neuroprotective in a porcine model of global ischemia-reperfusion in experimental cardiac arrest. However, no comprehensive analyses have been conducted at gene expression level.

**Methods:**

Pigs underwent either untreated cardiac arrest (CA) or CA with subsequent cardiopulmonary resuscitation (CPR) accompanied with an infusion of saline or an infusion of saline with MB. Genome-wide transcriptional profiling using the Affymetrix porcine microarray was performed to 1) gain understanding of delayed neuronal death initiation in porcine brain during ischemia and after 30, 60 and 180 min following reperfusion, and 2) identify the mechanisms behind the neuroprotective effect of MB after ischemic injury (at 30, 60 and 180 min).

**Results:**

Our results show that restoration of spontaneous circulation (ROSC) induces major transcriptional changes related to stress response, inflammation, apoptosis and even cytoprotection. In contrast, the untreated ischemic and anoxic insult affected only few genes mainly involved in intra-/extracellular ionic balance. Furthermore, our data show that the neuroprotective role of MB is diverse and fulfilled by regulation of the expression of soluble guanylate cyclase and biological processes accountable for inhibition of apoptosis, modulation of stress response, neurogenesis and neuroprotection.

**Conclusions:**

Our results support that MB could be a valuable intervention and should be investigated as a therapeutic agent against neural damage associated with I/R injury induced by cardiac arrest.

## Background

Despite recent advances in out-of-hospital cardiac arrest (CA) resuscitation, hypoxic-ischemic brain damage still causes considerable mortality and morbidity. Of the patients who survive to discharge, only 20% or fewer will have good neurologic function at the end of 1 year [1] After successful CPR and restoration of spontaneous circulation (ROSC) neuronal death initiated by ischemia during CA is increased also during reperfusion leading to secondary postischemic-anoxic encephalopathy [2], part of the so-called postresuscitation syndrome [3,4]. Cerebral recovery is dependent on duration of arrest and cardiopulmonary resuscitation (CPR), and numerous factors related to basic, advanced, and prolonged life support [5,6]. Except for the use of mild hypothermia after ventricular fibrillation cardiac arrest, currently recommended therapy in the 2005 guidelines of the European Resuscitation Council [7], clinical neuroprotection practice rests solely on extrapolation from animal experimental work or weak clinical studies[8]. In recent years, especially in critical care research, swine have increasingly been employed because they share similar cardiovascular and physiologic properties with humans [9,10]. Furthermore, porcine brains, like human, are gyrencephalic which make them closely related to the human brain in structure and function. Accordingly, the response of pigs to disease and treatment are often comparable to that of human [11] and data concerning pig brain anatomy and neurochemistry have increased considerably in recent years [12,13].

Safety/FDA approved methylene Blue (MB), a cationic thiazine dye with a low toxicity profile at low doses, is efficiently trapped in the brain and its concentration is over 10 times higher in the brain than in the circulation one hour after systemic administration [14], indicating a rapid and extensive accumulation in the nervous system. MB has been used as a neuroprotective agent in drug-induced encephalopathy, dementia and manic-depressive psychosis [15-17]. Furthermore, MB exhibits promising cardio- and neuroprotective properties in experimental cardiac arrest [18,19] and is effective in both attenuating ischemia-reperfusion (I/R) syndrome [20] and increasing short-term survival after resuscitation from cardiac arrest [21].

In order to gain further understanding of the mechanisms behind the neuroprotective effects of MB, we conducted a microarray analysis of the changes in gene expression in the ischemic brain in our pig model of experimental cardiac arrest and subsequent ischemia-reperfusion injury.

## Methods

Animal treatment and experimental procedures were approved by the Uppsala Institutional Review Board for Animal Experimentation. The piglets were handled according to the guidelines of the Swedish National Board for Laboratory Animals and the European Convention of Animal Care. Anaesthesia was used in all surgical interventions.

### Animals

Experiments were performed on 30 piglets of Swedish triple breed of both sexes aged 12-14 weeks and with a mean weight of 25.8 kg ± 1.3 kg.

### Cardiac arrest model

**Figure 1:**
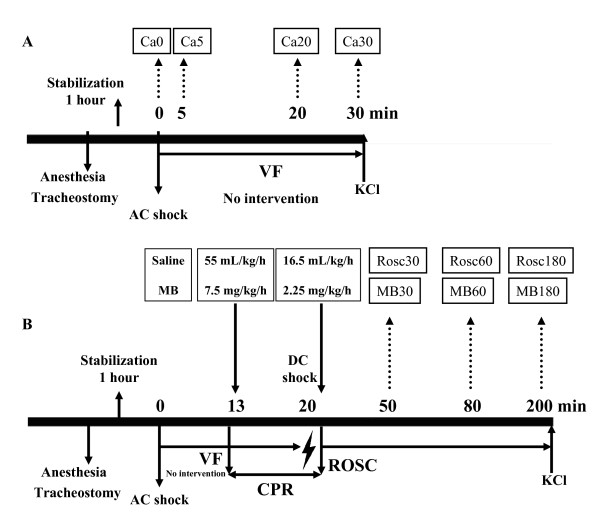
**Experimental procedure**. After stabilization for 1 h, animals were subjected to ventricular fibrillation. They were either sacrificed with a potassium chloride injection (KCl) after 0, 5, 20 or 30 min after untreated cardiac arrest (A) or, after 12 min of cardiac arrest and 8 min of cardiopulmonary resuscitation (CPR), return of spontaneous circulation (ROSC) was carried out and animals received a saline solution (Rosc groups) or methylene blue (MB groups) before sacrifice at 30, 60 and 180 min post-resuscitation (B).

Previous use of the same model of extended cardiac arrest (12 min of untreated cardiac arrest (CA) and 8 min of cardiopulmonary resuscitation (CPR) has already been described [19]. Here we used the same anaesthesia, fluid administration surgical preparation and experimental protocol to induce CA. Return of spontaneous circulation and post-resuscitative treatments either with normal saline or with MB were administered following the protocol previously described by our group [18]. After completion of study, all animals received an injection of 10 mL potassium chloride 20 mmol/mL and were sacrificed. In all the animals the skull was opened in prone position immediately before death and the brain was taken out and frozen in liquid nitrogen within 1 min after death. Thirty pigs were divided into three groups. The first group (CA, n = 12) underwent untreated CA. The brain of the animals was removed either immediately after CA (Ca0 n = 3), or after 5 min (Ca5 n = 3), 20 min (Ca20 n = 3) and 30 min (Ca30 n = 3) respectively (Figure 1A). The other two groups underwent CA followed by CPR and received either an infusion saline (ROSC n = 9) or an infusion of MB (10 mg/mL Metyltioninklorid, Apoteket, Umeå, Sweden) with saline (MB n = 9) as described in Figure 1B. Brain samples from resuscitated animals were collected at 30 min (Rosc30 and MB30 groups), 60 min (Rosc60 and MB60 groups) or 180 min (Rosc180 and MB180 groups) after ROSC.

### RNA extraction

Total RNA was extracted using RNeasy Lipid Tissue kit (Qiagen)according to the manufacturer's instructions. Contaminating genomic DNA was removed during RNA purification by addition of an on-column DNase digestion step with RNase-Free Dnase I (Qiagen). RNA quality was verified on an Agilent 2100 Bioanalyzer (Agilent).

### Affymetrix gene profiling and microarray analysis

Total RNA (10 μg) was first converted to double-stranded cDNA (One-cycle cDNA synthesis kit, Affymetrix UK Ltd) and then to biotinylated cRNA (IVT labelling kit, Affymetrix UK Ltd) as described in the One-cycle Target Labelling protocol (Affymetrix UK Ltd). After fragmentation and quality confirmation (Agilent 2100 Bioanalyzer), 10 μg of the biotinylated cRNA were hybridized for 16 hours to an Affymetrix GeneChip Porcine Genome Array. The array was then washed and stained with streptavidin-phycoerythrin solution using the Fluidics Station 450 (Affymetrix UK Ltd.) before it was scanned using the Affymetrix GeneChip Scanner 3000. Data were normalized and comparative analysis results were obtained from Affymetrix GeneChip Operating Software (GCOS v1.4; Affymetrix UK Ltd). The results were deposited to GEO database under accession number GSE22165. Statistical analysis was performed using the Significance Analysis of Microarrays (SAM) statistics program to determine genes that were significantly regulated at each time point [22]. SAM uses a permutation method to determine the significance of changes in expression by assimilating a set of gene-specific t-tests and hence reports a false-discovery rate (FDR) rather than a probability. Data query, mining, filtering and sorting as well as hierarchical cluster analysis and principle component analysis (PCA) were performed using Spotfire DecisionSite^® ^for Functional Genomics software (Tibco-Spotfire Inc., Cambridge, MA, USA).

### Gene filtering and selection

In a first step, after normalization, results from the GeneChip Operating Software (GCOS) were considered. Genes with raw intensity values that did not meet a minimum threshold (more than 30 in at least one of the samples) or described as absent in all 30 samples were excluded from the results. The selected probe sets were then submitted to statistical analysis to identify significant changes in gene expression. Pair-wise comparison analysis based on GCOS statistical algorithm was carried out to compare animals of the experimental group to each animal of the corresponding control group. The group means were also compared using the SAM software. By combining these 2 approaches, we obtained a list of modulated genes for each condition. The stringent criteria for the lists of genes were that the FDR values obtained with the SAM software were inferior to 5% and there were 9 significant change calls generated by GCOS. Furthermore, the lists of genes were restricted to include only genes for which expression changes were ≥ ± 1.5-fold. Changes which occurred 5, 20 or 30 min after cardiac arrest or 30, 60 or 180 min after ROSC were analyzed using the Ca0 as control group. Changes after 30, 60 or 180 min of MB treatment following ROSC were analyzed using the Rosc30, Rosc60 and Rosc180 as control groups, respectively.

### Confirmatory Real-Time PCR analysis

Data generated from microarrays were confirmed using real-time PCR (qPCR).

RNA was extracted as described above and then was reversely transcribed using oligo(dT) and random hexamers and iScript reverse transcriptase (Bio-Rad Laboratories, USA). The qPCR was conducted on the MyiQ single-colour Real-time PCR detection system (Bio-Rad Laboratories, USA). Each reaction contained 12.5 μl 2× IQ SYBR Green Supermix (Bio-Rad Laboratories, USA), primers and template to total volume of 25 μl. The thermal profile used for amplification was 95°C for 5 min followed by 40 cycles of 97°C for 15 sec, 60°C for 15 sec, and 72°C for 15 sec. At the end of the amplification phase, a melting-curve analysis was carried out on the products formed. All primers were designed by Beacon Designer 5.10 (Biosoft International, Palo Alto, CA). Table 1 summarizes the information about the primers, including Genbank accession numbers.

**Table 1:** Sequences of primers used for qRT-PCR

Gene symbol	Primer sequence (5'->3')	Product	GenBank Acc*
*sdha*	Forward: ACTCGCTCCTGGACCTCGTTG	158 bp	DQ845177
	Reverse: CCTTATGGTTCCGTTCGCAAATCTC		
*Hsp22*	Forward: AGCCCTGGAAAGTGTGTGTC	198 bp	AY609863
	Reverse: GGGAAAGCGAGGCAAATACTG		
*Hsp27*	Forward: AGGAGCGGCAGGATGAG	100 bp	AY574049
	Reverse: GACAGGGAGGAGGAGACC		
*Hsp40*	Forward: GACCAGACCTCCAACAACATTC	189 bp	AF288820
	Reverse: ATCTTTGAACACAACGGGTATGG		
*Hsp70*	Forward: GCTCAGTGGCATACCTC	182 bp	X68213
	Reverse: CACGAACCATCCTCTCC		
*Hsp72*	Forward: CAACAAGATCACCATCAC	79 bp	AY466608
	Reverse: CTTCTCCGCCTCCTG		
*Hsp90*	Forward: AACCGCTCTGCTGTCTTC	103 bp	U94395
	Reverse: GTCGTCCTCATCAATACCAAG		
Similar to *dusp1*	Forward: CAACCACAAGGCGGACATC	200 bp	AK232967
	Reverse: TGCTCCTCCTCTGCTTCAC		
Similar to *ddit4*	Forward: AGTAAGACAGTTAAGTCAACAGTG	91 bp	XM_001925275
	Reverse: GCAGCGAGCACACATCC		
Similar to *gadd45b*	Forward: GCTGCGATCTGAAGGTC	102 bp	AK231760
	Reverse: GCCACACGACAGTTCC		
Similar to *ddit3*	Forward: GAACGAACGGCTCAAG	182 bp	CK454369
	Reverse: TGGCACTGGTAAGAAGG		
*rgs2*	Forward: CCGCCGCAGATCACCACAGAG	133 bp	DQ150111
	Reverse: CCTGGCTTCCTGACTCACTAACTCC		
Similar to *nfil3*	Forward: GCCCGATCCACTCTCC	112 bp	XM_001928870
	Reverse: ATGGCTTTGGCTTTAATCC		
Similar to *set*	Forward: TGCCTGCCACCACCATC	138 bp	BQ598788
	Reverse: CCACCAACACGGACTTCTTAC		
*cart*	Forward: CTGCTGCTGCTGCTAC	177 bp	AF338229
	Reverse: CTTCTCATAAATCGGGATACG		
Similar to *dhcr24*	Forward: AGTGTATGGTGTGTG	116 bp	CF360734
	Reverse: GTTCTGGACAGTAGG		
*ndrg2*	Forward: CCCGTGTTCCCTTTGG	151 bp	DQ985169
	Reverse: GGTGACTAAGAGCATATCG		

*GeneBank accession number at www.ncbi.nlm.nih.gov

All experiments were performed in triplicates for every sample and contained negative controls. Results were evaluated by MyiQ single-colour Real-time PCR detection system software (Bio-Rad Laboratories, USA). The expression levels for *Hsp22*, *Hsp27*, *Hsp40*, *Hsp70*, *Hsp72*, *Hsp90*, *rgs2*, *cart*, *ndrg2 *as well genes similar to *dusp1*, *ddit3*, *gadd45b*, *nfil3*, *set *and *dhcr2*4 mRNA transcripts were described as the ratios of the targets normalized to the endogenous reference (*sdha*) and then the ratios for fold change relative to the calibrator were obtained. The calibrator was constituted from brain samples of piglets belonging to the Ca0 group.

## Results

### Gene annotation

The Affymetrix porcine genome microarray is minimally annotated. Approximately 11% of the probe sets on this array are described with gene names, posing a challenge to biological interpretation of data. Lack of annotation is likely due to the limited availability of full-length porcine cDNA sequence. To gain further information about the identity and/or function of the selected sequences which were not associated to a protein name, BLAST sequence-similarity searches were performed. After careful verification of the resulting alignments, probe sets sharing sequence similarity with known proteins from other species were annotated as "similar to". To avoid redundancy, only gene symbols are mentioned in the text while full names are used in the figures and tables. Furthermore genes annotated "similar to" will be considered as sharing the same function as the gene they were associated with.

### Gene expression profiles after untreated CA

We identified 28 transcripts as differentially expressed, of which, 21 up-regulated and 7 down-regulated (Table 2). Only 1 gene, similar to *SLC30A10 *(a zinc transporter) showed a constant increase (4-fold) from 5 min to 30 min. Another zinc transporter, *SLC30A9 *was found modestly increased at 30 min (1.5-fold). The other transcripts were found significantly changed only at 1 time point with the exception of *PITRM1 *which was decreased at 5 and 20 min and 2 uncharacterized genes which were up and down-regulated (both at 20 and 30 min), respectively. Although most of the regulated transcripts showed only moderate changes, prealbumin, *GNAL *and beta-neoendorphin were highly up-regulated at 20 min (more the 10-fold). Even though this data set was too small to be submitted to advanced functional analyses, a functional category could be assigned for each probe set (see Table 2).

**Table 2:** Differentially expressed genes after 5 min (Ca5), 20 min (Ca20) and 30 min (ca30) of untreated CA

Regulation	Known Function	Probe Set	Gene Products	Fold change		
				
				Ca5	Ca20	Ca30
Up	Transcription	Ssc.9200.1.A1_at	similar to *ZCCHC12*, Zinc finger CCHC domain-containing protein 12	1.3	2.1*	1.3
	Transcription	Ssc.2132.1.S1_a_at	similar to *RPS6KA5 *ribosomal protein S6 kinase alpha-5	0.8	2.3*	1.0
	Transcription	Ssc.22527.1.A1_at	similar to homeobox protein *PBX3*, Pre-B-cell leukemia transcription factor 3.	0.9	2.5*	1.3
	Signal transduction	Ssc.14404.1.A1_at	*HTR2C*, 5-hydroxytryptamine (serotonin) receptor 2C	1.1	4.6*	1.5
	Transport	Ssc.640.1.S1_at	*TTR*, Transthyretin (Prealbumin)	27	2789*	2.6
	Cation transport	Ssc.3850.1.S1_at	similar to *NNAT*, neuronatin	1.4	1.9*	1.4
	Potassium transport	Ssc.6932.1.A1_at	similar to *DPP10*, inactive dipeptidyl peptidase 10	1.3	1.1	1.5*
	Zinc transport	Ssc.7040.1.A1_at	similar to *SLC30A9*, solute carrier family 30 member 9	1.4	1.1	1.5*
	Zinc transport	Ssc.17849.1.A1_at	similar to *SLC30A10*, solute carrier family 30 member 10	4.0*	4.5*	4.7*
	Calcium-mediated signalling	Ssc.29371.1.A1_at	similar to *GNAL *guanine nucleotide-binding protein G(olf) subunit alpha	1.6	16.5*	1.1
	Calcium-mediated signalling	Ssc.19549.1.S1_at	similar to *NECAB1*, Neuronal calcium-binding protein 1	1.5	1.0	2.0*
	Calcium-mediated signalling	Ssc.3649.1.A1_at	similar to *RCAN3 *regulator of calcineurin 3 (calcipressin-3)	1.1	1.5*	1.0
	Opioid peptide	Ssc.121.1.S1_at	*PDYN*, Beta-neoendorphin-dynorphin	6.2	14.2*	1.4
	Stress response	Ssc.7903.1.A1_at	similar to *HSPA13*, Heat shock 70 kDa protein 13 (stress 70 protein chaperone microsome-associated 60 kDa protein)	1.3	0.9	2.0*
	Neurogenesis	Ssc.19425.1.A1_at	similar to *MAPT*, microtubule-associated protein tau	1.7*	1.0	1.2
	Nucleic acid metabolism	Ssc.6238.2.S1_at	similar to *AK3*, GTP:AMP phosphotransferase mitochondrial (adenylate kinase 3)	1.3	1.1	1.7*
	Not determined	Ssc.12504.1.A1_at	similar to protein *FAM14A*	0.9	2.0*	0.9
	Not determined	Ssc.30063.1.A1_at	unknown	2.4*	1.0	1.0
	Not determined	Ssc.29510.1.A1_at	unknown	1.6	1.4	2.1*
	Not determined	Ssc.21096.1.S1_at	unknown	1.3	3.6*	4.5*
	Not determined	Ssc.31095.1.A1_at	unknown	1.3	2.0*	0.9
						
Down	Transcription	Ssc.19152.1.S1_at	similar to *CPSF7*, cleavage and polyadenylation specificity factor 7	0.8	0.8	0.6*
	Signal transduction	Ssc.13752.1.S1_at	similar to *PIK3R1*, phosphatidylinositol 3-kinase p85 regulatory subunit alpha	0.7	0.3*	0.8
	Signal transduction	Ssc.11787.1.S1_at	similar to *RASSF2*, Ras association domain-containing protein 2	0.9	0.6*	0.8
	Proteolysis	Ssc.3063.1.S1_at	similar to *PITRM1*, pitrilysin metalloproteinase 1	0.6*	0.6*	0.9
	Glucose metabolism	Ssc.19476.2.A1_s_at	*PYGM*, Glycogen phosphorylase	0.7	0.8	0.6*
	Immune response	Ssc.13778.1.S1_at	IgG, Immunoglobulin G, heavy chain	0.5	0.8	0.1*
	Not determined	Ssc.17442.1.S1_at	unknown	0.9	0.6*	0.6*

### Gene expression profiles after ROSC

Transcriptional changes for Rosc 30, 60 and 180 were analyzed using Ca0 as baseline. For changes specifically induced by MB treatment, MB30, 60 and 180 groups were compared to their corresponding ROSC (with saline) controls i.e. Rosc 30, 60 and 180, respectively. After gene filtering, we obtained a final list of 518 probe sets showing significant changes of expression after either ROSC or MB treatment. Manual annotations gave a total of 317 probe sets (61%) similar to a known protein, 80 (16%) identified genes and 121 (23%) lacking any sequence similarity in any database and therefore called "unknown" (for a detailed list see Additional file 1).

**Figure 2:**
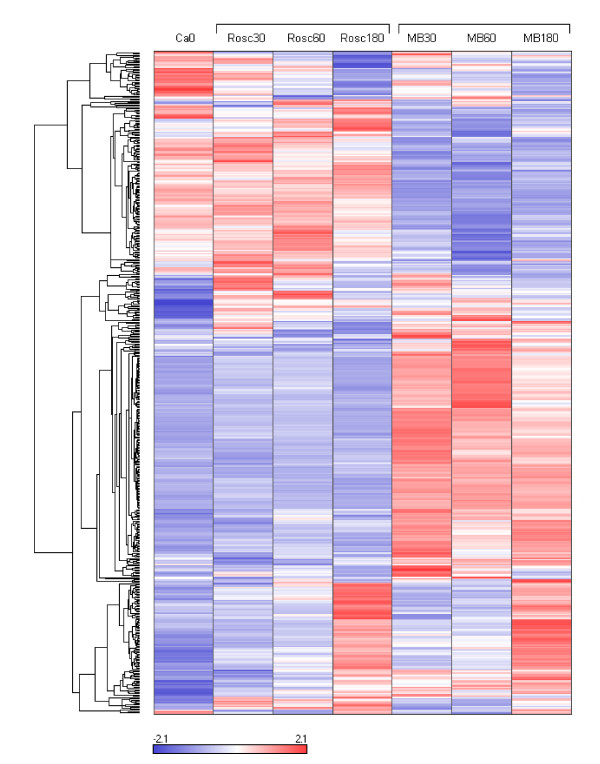
**Hierarchical cluster analysis of the 518 differentially expressed genes after ROSC with saline (Rosc groups) or MB (MB groups) treatment**. Each row represents a gene and each column the colour coded mean value of the three animals for each group. Genes with similar expression patterns are clustered together (dendrogram on the left).

**Figure 3:**
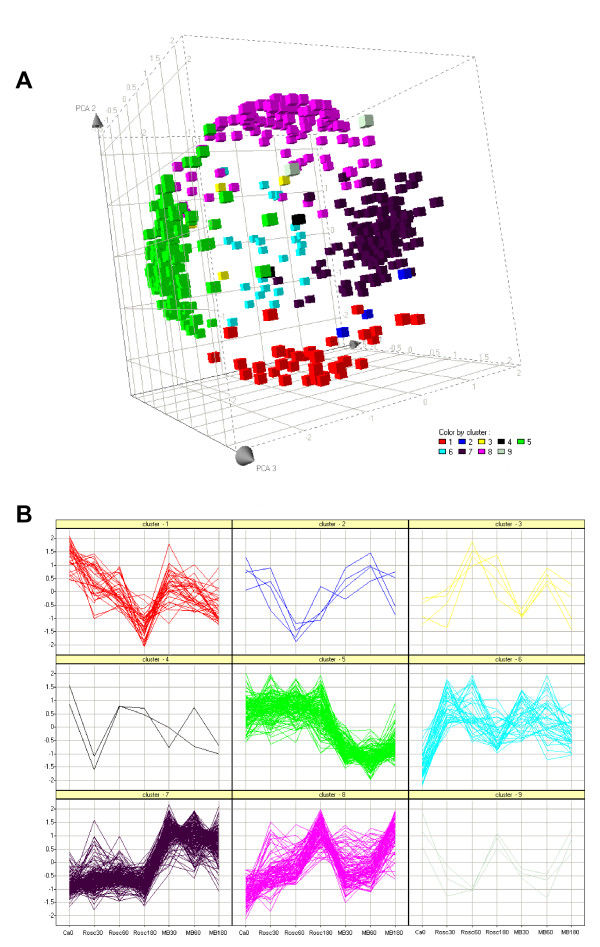
**Principle component analysis (A) and scatter plot of the expression profiles (B) defined by the 9 clusters generated by hierarchical clustering**. Each cluster can be easily identified by its associated colour.

**Figure 4:**
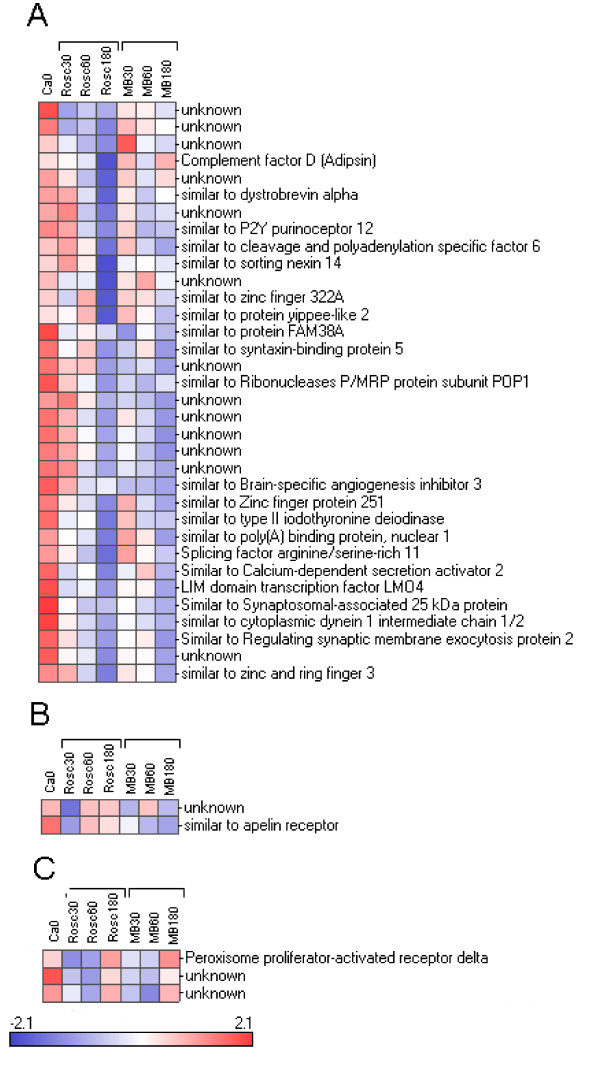
**Hierarchical clustering of three subsets of genes segregating in cluster 1 (A), "cluster" 4 (B) and "cluster" 9 (C) and for which expression levels decreased after ROSC**.

**Figure 5:**
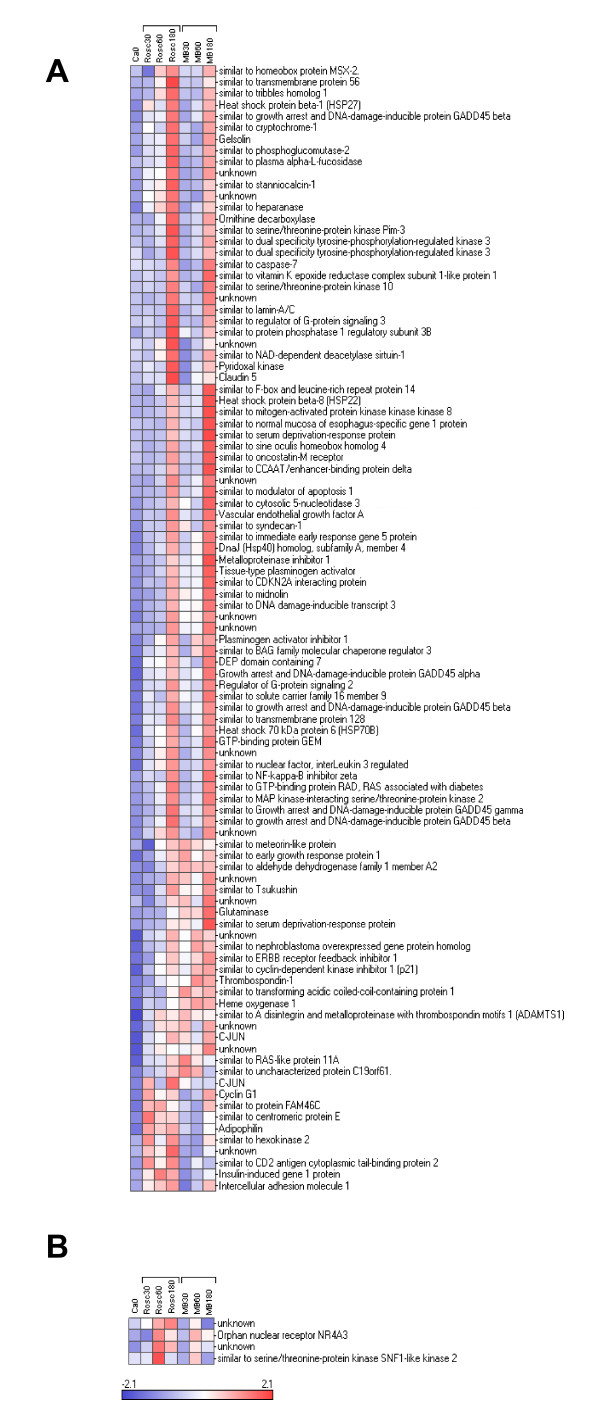
**Hierarchical clustering of two subsets of genes segregating in cluster 8 (A), "cluster" 3 (B) and for which expression levels increased after ROSC**.

**Figure 6:**
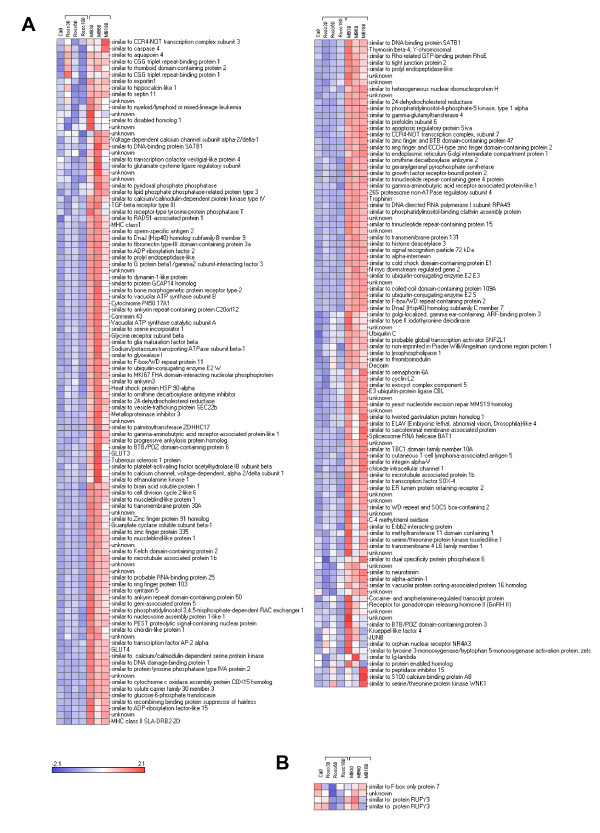
**Hierarchical clustering of two subsets of genes segregating in cluster 7 (A), "cluster" 2 (B) and for which expression levels were induced by MB**.

**Figure 7:**
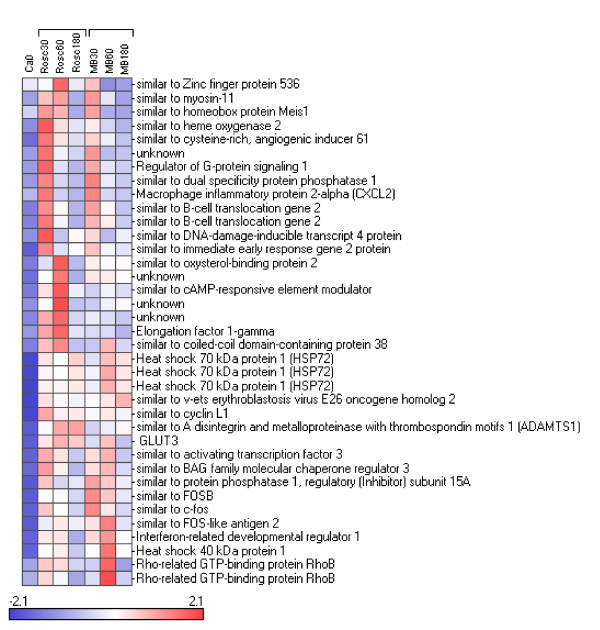
**Hierarchical clustering of a subset of genes segregating in cluster 6 and for which expression levels were early induced after ROSC**.

**Figure 8:**
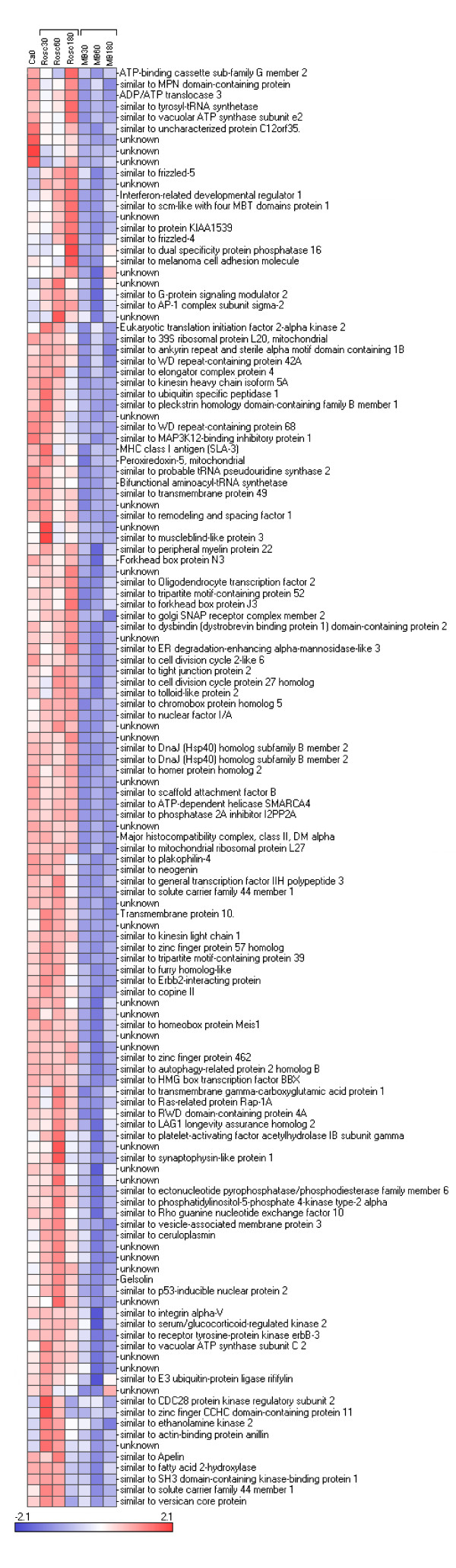
**Hierarchical clustering of a subset of genes segregating in cluster 5 and for which expression levels were early decreased after MB treatment**.

**Figure 9:**
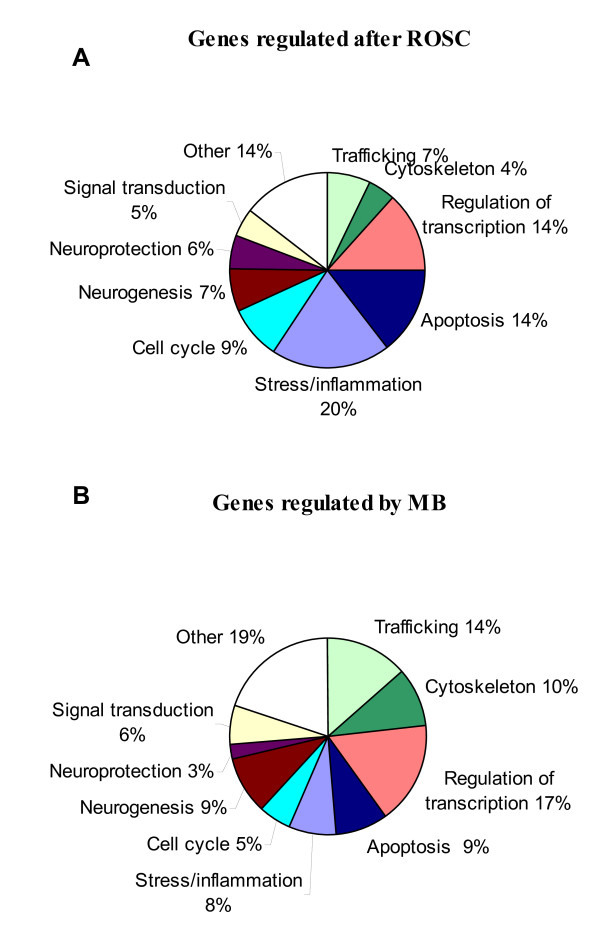
**Functional analysis of genes regulated after ROSC (A) and genes affected by MB treatment (B)**. Proportion of differentially expressed genes in different functional categories. One gene can be assigned several functions and therefore belong to more than one category.

We reorganized this large data set by performing hierarchical clustering (see Figure 2). The data set was split into 9 parts named cluster 1 to 9 varying in size from 2 to 207 genes. The relevance and the relationship between the clusters was studied using PCA (Figure 3A) and expression patterns (Figure 3B). The results showed 5 distinct subsets of transcripts named as cluster 1, 5, 6, 7 and 8 which contained 34, 130, 41, 205 and 99 genes, respectively. Four more diffuse and heterogeneous very small subsets (4 members or less) were not considered as valid clusters. Therefore their members were associated to the main cluster they appeared to be most similar to, based on the PCA 3D representation. "Clusters" 4 (2 genes) and 9 (3 genes) were grouped with cluster 1 (Figure 4), "cluster" 3 (4 genes) with cluster 8 (Figure 5) and "cluster" 2 (4 genes) with cluster 7 (Figure 6) while cluster 6 (Figure 7) and cluster 5 (Figure 8) remained unaffected. Relationships between the main clusters were clearly displayed by the PCA analysis: cluster 8, corresponding to genes up-regulated after ROSC, opposite to cluster 1 (down-regulated after ROSC) and cluster 7, corresponding to genes induced by MB, opposite to cluster 5 (decreased by MB) (see Figure 3A). Cluster 6 represented transcripts with an early increase of expression after ROSC and was standing by itself. Annotated genes were classified by functional category. The results for differentially expressed proteins after ROSC and by MB treatment, respectively, were summarized in Figure 9. A search for overrepresented functional classes within each cluster showed significant results only for the group formed by cluster 8. In this group of transcripts induced after ROSC, 6 functional classes corresponding to response to stress (p < 0.001), regulation of transferase activity (p = 0.001), regulation of protein kinase activity (p = 0.001), apoptosis/type I programmed cell death (p = 0.034), activation of mitogen-activated protein (MAP) kinase kinase kinase (p = 0.044) and programmed cell death (p = 0.044) were overrepresented. A list of the processes affected after ROSC and/or by MB (addressed in the discussion) can be found in Additional file 2.

### Replication of gene expression profiles by RT-qPCR

**Figure 10:**
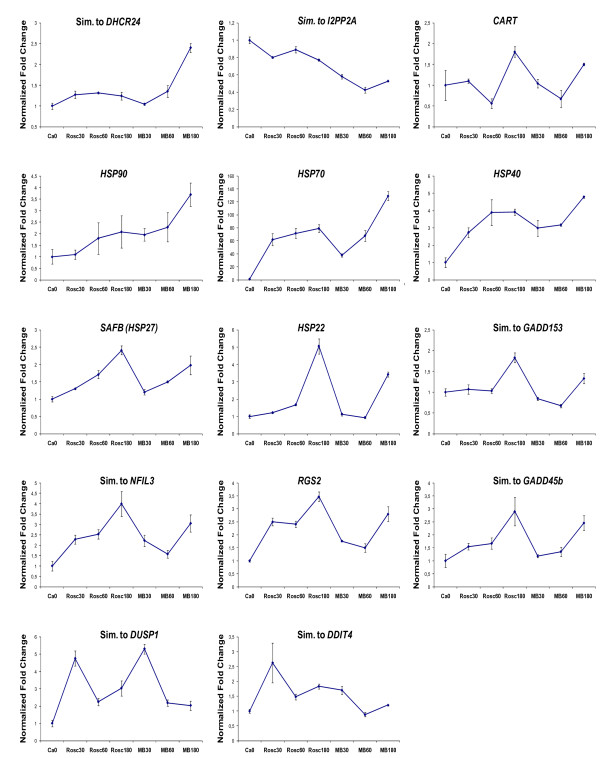
**Real-time qPCR analysis of genes that showed a significant differential expression in the microarray study**. The genes were selected based on relevance to ischemia/reperfusion injury pathophysiology. Data are shown as means (fold change) of normalized ratios of three biological replicas (y-axis). Error bars represent standard error of the mean (SEM).

Validation of differentially expressed genes according to the microarray results was conducted by RT-qPCR. We selected genes with different expression patterns and the results showed a good correlation with the findings from microarrays (Figure 10)

## Discussion

### Effects of cardiac arrest/global ischemia

Interruption of blood flow after CA limits the delivery of oxygen and glucose to neurons causing ATP reduction which has numerous consequences including neurotoxicity, DNA damage, and production of free radicals [23]. In our model, we found only 28 significantly differentially expressed transcripts in total after global ischemia. Among the moderately up-regulated genes, it is noteworthy that 7 transcripts affected by ischemia belonged to ion transport and calcium signalling functions as it is well known that one of the first consequences of ischemia-induced apoptosis is a disruption of ionic gradients across membranes with increase in extracellular potassium and influx of sodium, chloride and calcium into the cells. Interestingly our list included 2 zinc transporters: SLC30A9 (ZNT9) and SLC30A10 (ZNT10). These findings are in line with an earlier study showing that another member of the SLC30 family, ZNT1, was induced after transient forebrain ischemia in the Gerbil [24]. The SLC30 proteins are CDF (cation diffusion facilitatator) proteins and plasma membrane efflux transporters [25]. The mechanism of transport for many CDF proteins appears to be via zinc/H + or K + antiports. Therefore, despite their name, CDF proteins do not serve as diffusion facilitators but rather as secondary active transporters, using the gradient of other ions to drive the transport of zinc [26]. Identification of ZNT9 and ZNT10 as ischemia-induced genes adds therefore to the growing evidence suggesting that the role of zinc efflux transporters in cellular zinc homeostasis may be particularly important in postischemic neuronal death where high levels of zinc are accumulated by neurons [27].

### Effects of ROSC/reperfusion

Cardiac arrest and CPR elicit global brain ischemia and reperfusion causing immediate and delayed cell death. I/R injury results in oxidative stress leading to further generation of free radicals. This in turn activates numerous major signalling pathways, in particular through changes in gene expression which influence the survival or death of the cells.

The use of microarrays has been applied to study the effects of permanent or transient ischemia on total brain or distinct regions in rodent models but, to our knowledge, this is the first time that a genome-wide transcript profiling study is undertaken in porcine brain to study the effect of I/R. Our results showed that 141 genes were induced and only 39 were repressed after ROSC. Such an imbalance between up- and down-regulated genes could be due to a technical bias. Yet it is in agreement with a recent report where the authors monitored the genomic response to global ischemia and reperfusion in the rat brain and concluded that the ischemia/reperfusion response of the rat brain is therefore an active process [28]. A functional analysis of the clusters revealed that apoptosis/programmed cell death and stress response (34% of all induced genes) were overrepresented among the genes up-regulated after ROSC. Among them we found proteins belonging to the caspase-dependent apoptotic pathway, p53 and mitogen-activated protein kinase pathways, playing a pivotal role in stress and apoptosis. Consistent with findings in rodent, heat shock proteins are among the most prominently upregulated genes after the ischemic insult [29,30]. Overall our gene lists are in line with previous microarray studies monitoring cerebral ischemic insult [28-34] including proteins such as ICAM-1, a main actor for the regulation of ischemia-induced injury since its inhibition induces neuroprotection after I/R injury [35,36]; VEGFA as a main indicator for brain ischemia [37]; IFRD1, an early gene stimulating p53 and up-regulating iNOS after oxidative stress [38]; inducible HMOX1 and constitutive HMOX2, two rate-limiting enzymes for the catabolism of the prooxidant heme [39,40] and CHOP. a main marker of ER stress [41,42].

### Effects of MB treatment

One of the responses to cerebral ischemia is an increase in the production of NO, catalyzed by nitric oxide synthase (NOS) expression. There has been increasing evidence that neuronal nitric oxide synthase (nNOS)-induced NO is an important mediator in ischemic brain injury [43]. Methylene blue (MB) exerts an antioxidant effect by binding and inactivating the action of NO [44], blocking the soluble guanylate cyclase [45] - the primary receptor for NO - as well as inhibiting Nitric Oxide Synthase (NOS) [46]. Although transcriptional levels of NOS did not show any changes after treatment by MB in our study, we have previously reported that NOS enzymes were regulated by MB at the protein level, in porcine brain, already after 30 min (Miclescu et al., submitted). These results concur with the conclusion that NOS enzymes are preferentially regulated post-transcriptionally, thus allowing a more rapid adaptation of the enzyme activity to cellular stress variations [47]. As could be expected, we identified the sGC transcript in the set of genes regulated by MB together with HSP90, a chaperone forming a complex with both sGC and endothelial nitric oxide synthase (eNOS) for the modulation of NO production [48]. Although the modulation of cGMP/NO pathway is considered the most significant effect of MB, recent studies indicate that it has multiple cellular and molecular targets.

#### Inhibition of apoptosis

A number of anti-apoptotic genes were found stimulated by MB. Some of them exert their effect via inhibition of caspases: DHCR24 (seladin-1), which protects cells from oxidative stress by reducing caspase 3 activity [49,50]; PIP5K1, decreasing activated caspases [51]; ELAVL4, an actor in both neuron-specific RNA processing (for example protecting p21 mRNA from decay) and inhibition of mitochondria-dependent apoptosis [52,53]. Others were identified as antagonists of the MAPK/p53 apoptotic pathways: RND3, a pro-survival p53 target gene that inhibits ROCK I-mediated apoptosis in response to stress and participates to neurite extension [54]; CSDE1, which decreases p53/GADD45g/caspase3 transcription [55]; DDB1, a pro-survival p53 target involved in protein degradation [56]; DUSP6, a MAPK phosphatase which inactivates ERK [57]; YWHAZ, which supports cell survival by antagonizing apoptotic proteins and has a neuroprotective role as p38 MAPK inhibitor [58,59]; as well as CAMK4, a key protein involved in oxidative stress which promotes neuronal survival and inhibits apoptosis [60]. Although the anti-apoptotic effect of MB appeared to be mostly caused by stimulation of specific transcripts, the repression of the following genes participated to the overall protective effect of MB: SMARCA4, a chromatin remodelling factor and an apoptotic p53 target [61]; NEO which acquires its pro-apoptotic activity when cleaved by caspases [62]; TMEM49, a stress-induced protein that promotes formation of intracellular vacuoles involved in autophagy [63]; SET (I2PP2A) mediating neuronal apoptosis [64]; SLC25A6 which participates in the formation of the permeability transition pore complex (PTPC) responsible for the release of mitochondrial signals [65]; and SAFB, an apoptosis marker [66].

#### ER stress

Upon reperfusion after even a short period of transient global cerebral ischemia, protein synthesis is severely suppressed [67,68]. Ischemia-induced suppression of protein synthesis, which is initiated by activation of kinases phosphorylating the initiation factor 2 (eIF2a), has been attributed to ER dysfunction [69]. Our results showed that eIF2AK2 (PKR) one of the kinases that specifically phosphorylates eIF2a and therefore inhibits translation [70], was repressed by MB together with EDEM3, a protein enhancing the ER-associated degradation (ERAD) [71]. In addition, DNAJB9, an ER chaperone suppressing cell death and induced by ER stress [72] was also induced by MB. These results suggest a potential counteracting effect of MB on the shutdown of translation by ER stress.

#### Vesicle transport

Disruption of the cytoskeleton induced by apoptosis causes a depolymerization of microtubules followed by Golgi fragmentation and secretory membrane traffic arrest [73,74]. In the Golgi network, proteins are sorted and segregated into transport vesicles to be targeted to subcellular compartments or released such as neurotransmitters. Our observations suggested that vesicle trafficking might be affected by MB since proteins which mediate transport between ER and Golgi such as SEC22b, STX5, KDELR2 and ERGIC1 [75,76,78] and VPS16 and GGA3 between Golgi and the endosomes [79,80] were up-regulated. On the other hand, proteins such as: VAMP3, a vesicle-associated membrane protein involved in regulating membrane traffic; AP1S2, modulating the dynamics of the cytoskeleton [81]; and GOSR2 involved in transport of proteins from the cis/medial-Golgi to the trans-Golgi network [75]; were down-regulated by MB.

#### Neurogenesis

Microtubules, a major component of the cytoskeleton which is damaged by ischemia, are crucial for intracellular transport and neurogenesis/neural regeneration. For example, the principle mechanisms involved in neurite extension and axonal pathfinding rely upon the reorganization of cytoskeletal elements induced by microtubule-associated proteins (MAPs) [82]. Our results showed changes in a number of microtubule-related proteins induced by MB: GABARAPL1 and MAP1B, two members of the MAP family; EXOC5 mediating the targeting of post-Golgi secretory vesicles and stimulating neurite outgrowth [83]; NIPA1 regulating synaptic growth and axonal microtubules [84]; INA, an intermediate filament protein involved in the morphogenesis of neurons; CAMK4 and SEMA6A, both involved in regulation of microtubule dynamics [85-87]; and SEPT11, which regulates microtubule stability through interaction with MAP4 [88]. In contrast, 2 microtubule subunits, the kinesins KIF5A and KLC1, were inhibited by MB. In addition to stimulation of the microtubule networks, MB induced proteins important for brain repair or regeneration such as: BASP1, TM4SF1, NT, SMARCA1 and RND3, which all promote neurite outgrowth [54,89-92]; RUFY3, a pro-survival gene implicated in the formation of a single axon in developing neurons [93]; GMFB which stimulates neural regeneration; SC4MOL, involved in brain repair [94]; CASK which plays a role in synaptogenesis [95]; as well as PAFAH1B2, DAB1 and PREX1 involved in brain cell migration [96-98]. The effect of MB on neurogenesis included also the inhibition of OLIG2, a repressor of neurogenesis [99] and two axon-guidance regulating proteins, NEO and NFIA [62,100].

#### Neuroprotection

MB induced genes known to have a neuroprotective effect such as: CX43, the primary component protein in astrocytic gap junctions playing multiple roles in the mitigation of apoptotic neuronal damage in cerebral ischemia[101,102];. THBD, which activates the anti-inflammatory and cytoprotective activated protein C (APC) thus leading to promotion of neovascularization and neurogenesis in the post-ischemic brain [103]; AQP4, a neuroprotective water-specific channel, osmoreceptor regulating water flow in central nervous system [104,105]; and CART, an intriguing peptide which protects brain from damage both through ERK activation in ischemic stroke [106] and potential preservation of mitochondrial function and prevention of energy failure after I/R injury [107].

## Conclusions

All these findings suggest that MB exerts neuroprotection by regulation of the expression of soluble guanylate cyclase and diverse biological processes from inhibition of apoptosis and reversal of the shutdown of translation to restoration of functional cellular trafficking and activation of brain repair/regeneration genes as well as induction of critical neuroprotective proteins. Therefore they support that MB could be a valuable intervention and should be investigated as a therapeutic agent against neural damage associated with I/R injury induced by cardiac arrest.

## Competing interests

The authors declare that they have no competing interests.

## Authors' contributions

CM performed the experimental work and drafted the manuscript. LW performed the surgery on the animals and participated in experimental design and data evaluation. Both authors read and approved the final version of the manuscript.

## Pre-publication history

The pre-publication history for this paper can be accessed here:

http://www.biomedcentral.com/1755-8794/3/27/prepub

## Supplementary Material

Additional file 1 **Affymetrix probe set identifiers and gene symbols and annotations for all genes found significantly differentially expressed after return of spontaneous circulation and/or treatment with methylene blue**. Annotations are arranged in the order they appear in each cluster (from top to bottom). table. Click here for file

Additional file 2 **Processes and Proteins affected by ROSC and MB respectively**. table. Click here for file
