# Supplementary material for: Effect of methylene blue on the genomic response to reperfusion injury induced by cardiac arrest and cardiopulmonary resuscitation in porcine brain
Source: BMC Med Genomics. 2010 Jul 1;3:27. doi: 10.1186/1755-8794-3-27 (PMC2904268; doi:10.1186/1755-8794-3-27)
Supplement: Additional file 1 — Affymetrix probe set identifiers and gene symbols and annotations for all genes found significantly differentially expressed after return of spontaneous circulation and/or treatment with methylene blue. Annotations are arranged in the order they appear in each cluster (from top to bottom). table. [file 1755-8794-3-27-S1.PDF]

Additional file 1. Affymetrix probe set identifiers and gene symbols and annotations for all genes found significantly differentially expressed after return of spontaneous circulation and/or treatment with methylene blue.
Annotations are arranged in the order they appear in each cluster (from top to bottom).

Cluster 1

probe set	gene symbol	gene annotation
Ssc.26080.1.A1_at	---	unknown
Ssc.6542.1.S1_at	---	unknown
Ssc.26738.1.A1_at	---	unknown
Ssc.11074.1.S1_at	*CFD*	Complement factor D (Adipsin)
Ssc.13346.1.A1_at	---	unknown
Ssc.11706.2.A1_at	*DTNA*	similar to dystrobrevin alpha
Ssc.8133.1.A1_at	---	unknown
Ssc.8449.1.A1_at	*P2RY12*	similar to P2Y purinoceptor 12
Ssc.6333.1.A1_at	*CPSF6*	similar to cleavage and polyadenylation specific factor 6
Ssc.24784.1.S1_at	*SNX14*	similar to sorting nexin 14
Ssc.4597.1.A1_at	---	unknown
Ssc.18924.1.A1_at	*ZNF322A*	similar to zinc finger 322A
Ssc.30768.1.S1_at	*YPEL2*	similar to protein yippee-like 2
Ssc.3898.1.S1_at	*FAM38A*	similar to protein FAM38A
Ssc.27354.1.S1_at	*STXBP5*	similar to syntaxin-binding protein 5
Ssc.13632.1.A1_at	---	unknown
Ssc.23194.1.A1_at	*POP1*	similar to Ribonucleases P/MRP protein subunit POP1
Ssc.10552.1.A1_at	---	unknown
Ssc.19000.1.A1_at	---	unknown
Ssc.27414.1.A1_at	---	unknown
Ssc.20419.1.S1_at	---	unknown
Ssc.8260.1.A1_at	---	unknown
Ssc.24703.1.A1_at	*BAI3*	similar to Brain-specific angiogenesis inhibitor 3
Ssc.3080.1.A1_at	*ZNF251*	similar to Zinc finger protein 251
Ssc.13736.1.A1_at	*DIO2*	similar to type II iodothyronine deiodinase
Ssc.21219.1.S1_at	*PABPN1*	similar to poly(A) binding protein, nuclear 1
Ssc.8959.1.A1_at	*SFRS11*	Splicing factor arginine/serine-rich 11
Ssc.12933.1.A1_at	*CADPS2*	Similar to Calcium-dependent secretion activator 2
Ssc.9714.1.S1_at	*LMO4*	LIM domain transcription factor LMO4
Ssc.13474.1.A1_at	*SNSP25*	Similar to Synaptosomal-associated 25 kDa protein
Ssc.23632.1.S1_at	*DYNC1I2*	similar to cytoplasmic dynein 1 intermediate chain 1/2
Ssc.8492.1.A1_at	*RIMS2*	Similar to Regulating synaptic membrane exocytosis protein 2
Ssc.9663.1.A1_at	---	unknown
Ssc.23046.1.S1_at	*ZNRF3*	similar to zinc and ring finger 3

Cluster 2

probe set		gene annotation
Ssc.21651.1.S1_at	*FBXO7*	similar to F-box only protein 7
Ssc.3945.2.S1_at	---	unknown
Ssc.27365.1.S1_at	*RUFY3*	similar to protein RUFY3
Ssc.27365.2.S1_at	*RUFY3*	similar to protein RUFY3

Cluster 3

probe set		gene annotation
Ssc.28256.1.A1_at	---	unknown
Ssc.761.1.S1_at	*NR4A3*	Orphan nuclear receptor NR4A3
Ssc.25022.1.A1_at	---	unknown
Ssc.11729.1.A1_at	*SNF1LK2*	similar to serine/threonine-protein kinase SNF1-like kinase 2

Cluster 4

probe set		gene annotation
Ssc.8646.1.A1_at	---	unknown
Ssc.28818.2.S1_a_at	*APLNR*	similar to apelin receptor

Cluster 5

probe set		gene annotation
Ssc.25103.1.S1_at	*ABCG2*	ATP-binding cassette sub-family G member 2
Ssc.16530.1.S1_at	*MPND*	similar to MPN domain-containing protein
Ssc.16638.1.S1_at	*SLC25A6*	ADP/ATP translocase 3
Ssc.16687.1.S1_at	*YARS*	similar to tyrosyl-tRNA synthetase
Ssc.2102.1.S1_at	*ATP6V0E2*	similar to vacuolar ATP synthase subunit e2
Ssc.25441.2.S1_a_at	*C12orf35*	similar to uncharacterized protein C12orf35.
Ssc.17073.2.S1_at	---	unknown
Ssc.13392.1.A1_at	---	unknown
Ssc.18772.1.A1_at	---	unknown
Ssc.29483.1.A1_at	*FZD5*	similar to frizzled-5
Ssc.13693.1.A1_at	---	unknown
Ssc.21987.1.A1_at	*IFRD1*	Interferon-related developmental regulator 1
Ssc.17204.1.S1_at	*SFMBT1*	similar to scm-like with four MBT domains protein 1
Ssc.18347.1.A1_s_at	---	unknown

Ssc.27661.1.A1_at	*Protein KIAA1539*	similar to protein KIAA1539
Ssc.1093.2.A1_at	*FZD4*	similar to frizzled-4
Ssc.25400.1.S1_at	*DUSP16*	similar to dual specificity protein phosphatase 16
Ssc.10391.1.A1_at	*MCAM*	similar to melanoma cell adhesion molecule
Ssc.19046.1.A1_at	---	unknown
Ssc.10051.2.A1_at	---	unknown
Ssc.13626.2.A1_at	*GPSM2*	similar to G-protein signaling modulator 2
Ssc.4135.2.A1_at	*AP1S2*	similar to AP-1 complex subunit sigma-2
Ssc.13428.1.A1_at	---	unknown
Ssc.7274.1.A1_at	*EIF2AK2*	Eukaryotic translation initiation factor 2-alpha kinase 2
Ssc.18191.1.S1_at	*MRPL20*	similar to 39S ribosomal protein L20, mitochondrial
Ssc.5344.1.A1_at	*ANKS1B*	similar to ankyrin repeat and sterile alpha motif domain containing 1B
Ssc.19651.1.S1_at	*WDR42A*	similar to WD repeat-containing protein 42A
Ssc.2812.1.A1_at	*ELP4*	similar to elongator complex protein 4
Ssc.10198.1.A1_at	*KIF5A*	similar to kinesin heavy chain isoform 5A
Ssc.7866.1.A1_a_at	*USP1*	similar to ubiquitin specific peptidase 1
Ssc.4898.1.S1_at	*PLEKHB1*	similar to pleckstrin homology domain-containing family B member 1
Ssc.7976.1.A1_at	---	unknown
Ssc.24219.1.S1_at	*WDR68*	similar to WD repeat-containing protein 68
Ssc.7916.1.A1_at	*MBIP*	similar to MAP3K12-binding inhibitory protein 1
Ssc.13780.4.S1_x_at	*SLA-3*	MHC class I antigen (SLA-3)
Ssc.460.1.S1_at	*PRDX5*	Peroxiredoxin-5, mitochondrial
Ssc.7494.1.A1_at	*TRUB2*	similar to probable tRNA pseudouridine synthase 2
Ssc.7362.1.S1_at	*EPRS*	Bifunctional aminoacyl-tRNA synthetase
Ssc.2648.1.S1_at	*TMEM49*	similar to transmembrane protein 49
Ssc.2925.3.S1_a_at	---	unknown
Ssc.10185.2.A1_at	*RSF1*	similar to remodeling and spacing factor 1
Ssc.12877.1.A1_at	---	unknown
Ssc.29038.1.A1_at	*MBNL3*	similar to muscleblind-like protein 3
Ssc.11108.1.A1_at	*PMP22*	similar to peripheral myelin protein 22
Ssc.26309.1.A1_at	*FOXN3*	Forkhead box protein N3
Ssc.30845.1.A1_at	---	unknown
Ssc.8174.1.A1_at	*OLIG2*	similar to Oligodendrocyte transcription factor 2
Ssc.29729.1.A1_at	*TRIM52*	similar to tripartite motif-containing protein 52
Ssc.23986.1.S1_at	*FOXJ3*	similar to forkhead box protein J3
Ssc.28472.1.S1_at	*GOSR2*	similar to golgi SNAP receptor complex member 2
Ssc.15660.2.S1_a_at	*DBNDD2*	similar to dysbindin (dystrobrevin binding protein 1) domain-containing protein 2
Ssc.21538.1.S1_at	---	unknown
Ssc.11308.1.A1_at	*EDEM3*	similar to ER degradation-enhancing alpha-mannosidase-like 3
Ssc.7136.1.A1_at	*CDC2L6*	similar to cell division cycle 2-like 6
Ssc.16907.1.A1_at	*TJP2*	similar to tight junction protein 2
Ssc.21280.2.A1_at	*CDC27*	similar to cell division cycle protein 27 homolog
Ssc.5656.1.S1_at	*TLL2*	similar to tolloid-like protein 2
Ssc.28320.1.S1_at	*CBX5*	similar to chromobox protein homolog 5
Ssc.23810.3.A1_at	*NFIA*	similar to nuclear factor I/A
Ssc.8039.1.A1_at	---	unknown
Ssc.17478.1.A1_at	---	unknown
Ssc.1180.1.S1_at	*DNAJB2*	similar to DnaJ (Hsp40) homolog subfamily B member 2
Ssc.1180.1.S1_at	*DNAJB2*	similar to DnaJ (Hsp40) homolog subfamily B member 2
Ssc.5560.1.S1_at	*HOMER2*	similar to homer protein homolog 2
Ssc.5092.1.S1_at	---	unknown
Ssc.5535.1.S1_at	*SAFB*	similar to scaffold attachment factor B
Ssc.26993.1.S1_at	*SMARCA4*	similar to ATP-dependent helicase SMARCA4
Ssc.6071.1.S1_at	*SET*	similar to phosphatase 2A inhibitor I2PP2A
Ssc.29248.1.A1_at	---	unknown
Ssc.13777.2.S1_at	*SLA-DMA*	Major histocompatibility complex, class II, DM alpha
Ssc.21277.1.S1_at	*MRPL27*	similar to mitochondrial ribosomal protein L27
Ssc.4567.1.A1_at	*PKP4*	similar to plakophilin-4
Ssc.17433.1.S1_at	*NEO1*	similar to neogenin
Ssc.8825.1.A1_at	*GTF2H3*	similar to general transcription factor IIH polypeptide 3
Ssc.25584.1.S1_at	*SLC44A1*	similar to solute carrier family 44 member 1
Ssc.30426.1.A1_at	---	unknown
Ssc.585.2.S1_a_at	*TMEM10*	Transmembrane protein 10.
Ssc.30904.1.S1_at	---	unknown
Ssc.2401.1.A1_at	*KLC1*	similar to kinesin light chain 1
Ssc.18265.1.A1_at	*ZFP57*	similar to zinc finger protein 57 homolog
Ssc.28916.1.A1_at	*TRIM39*	similar to tripartite motif-containing protein 39
Ssc.14247.1.S1_at	*FRYL*	similar to furry homolog-like
Ssc.7633.1.A1_at	*ERBB2IP*	similar to Erbb2-interacting protein
Ssc.5428.1.A1_at	*CPNE2*	similar to copine II
Ssc.27248.1.S1_at	---	unknown
Ssc.27938.1.S1_at	---	unknown
Ssc.4100.2.S1_at	*MED1*	similar to homeobox protein Meis1
Ssc.16458.1.A1_at	---	unknown
Ssc.9953.1.A1_at	---	unknown
Ssc.11839.1.S1_at	*ZNF462*	similar to zinc finger protein 462
Ssc.17368.1.S1_at	*ATG2B*	similar to autophagy-related protein 2 homolog B
Ssc.3206.1.S1_at	*BBX*	similar to HMG box transcription factor BBX
Ssc.11284.1.A1_at	*PRRG1*	similar to transmembrane gamma-carboxyglutamic acid protein 1

Ssc.1330.1.A1_at	*RAP1A*	similar to Ras-related protein Rap-1A
Ssc.2855.1.S1_at	*RWDD4A*	similar to RWD domain-containing protein 4A
Ssc.865.2.A1_at	*LASS2*	similar to LAG1 longevity assurance homolog 2
Ssc.15655.1.S1_at	*PAFAH1B3*	similar to platelet-activating factor acetylhydrolase IB subunit gamma
Ssc.17942.1.A1_at	---	unknown
Ssc.1892.1.S1_at	*SYPL1*	similar to synaptophysin-like protein 1
Ssc.8176.1.A1_at	---	unknown
Ssc.14418.1.A1_at	---	unknown
Ssc.21302.1.A1_at	*ENPP6*	similar to ectonucleotide pyrophosphatase/phosphodiesterase family member 6
Ssc.3889.1.S1_at	*PIP4K2A*	similar to phosphatidylinositol-5-phosphate 4-kinase type-2 alpha
Ssc.9402.1.A1_at	*ARHGEF10*	similar to Rho guanine nucleotide exchange factor 10
Ssc.11107.1.S1_at	*VAMP3*	similar to vesicle-associated membrane protein 3
Ssc.10453.1.S1_at	*CP*	similar to ceruloplasmin
Ssc.5364.1.S1_at	---	unknown
Ssc.3918.1.S1_at	---	unknown
Ssc.14200.1.A1_at	---	unknown
Ssc.14246.1.S1_at	*GSN*	Gelsolin
Ssc.16937.1.A1_at	*TP53INP2*	similar to p53-inducible nuclear protein 2
Ssc.9958.1.A1_at	---	unknown
Ssc.9451.1.S1_at	*ITGAV*	similar to integrin alpha-V
Ssc.24957.1.S1_at	*SGK2*	similar to serum/glucocorticoid-regulated kinase 2
Ssc.4203.1.S1_at	*ERBB3*	similar to receptor tyrosine-protein kinase erbB-3
Ssc.31160.1.A1_s_at	*ATP6V1C2*	similar to vacuolar ATP synthase subunit C 2
Ssc.27043.1.A1_at	---	unknown
Ssc.8262.1.A1_at	---	unknown
Ssc.28059.1.A1_at	*RFFL*	similar to E3 ubiquitin-protein ligase rififylin
Ssc.30956.1.A1_s_at	---	unknown
Ssc.12229.1.S1_at	*CKS2*	similar to CDC28 protein kinase regulatory subunit 2
Ssc.25591.1.S1_at	*ZCCHC11*	similar to zinc finger CCHC domain-containing protein 11
Ssc.3730.1.S1_at	*ETNK2*	similar to ethanolamine kinase 2
Ssc.7621.1.A1_at	*ANLN*	similar to actin-binding protein anillin
Ssc.26947.1.S1_at	---	unknown
Ssc.3436.1.A1_at	*APLN*	similar to Apelin
Ssc.26577.1.S1_at	*FA2H*	similar to fatty acid 2-hydroxylase
Ssc.30088.1.A1_at	*SH3KBP1*	similar to SH3 domain-containing kinase-binding protein 1
Ssc.5192.1.S1_at	*SLC44A1*	similar to solute carrier family 44 member 1
Ssc.5663.1.S1_at	*VCAN*	similar to versican core protein

Cluster 6

probe set		gene annotation
Ssc.13333.1.A1_at	*ZNF536*	similar to Zinc finger protein 536
Ssc.10392.1.A1_at	*MYH11*	similar to myosin-11
Ssc.24864.1.A1_at	*MEIS1*	similar to homeobox protein Meis1
Ssc.10776.1.A1_at	*hmox2*	similar to heme oxygenase 2
Ssc.9720.1.A1_at	*CYR61*	similar to cysteine-rich, angiogenic inducer 61
Ssc.23609.1.A1_at	---	unknown
Ssc.16039.1.S1_at	*RGS1*	Regulator of G-protein signaling 1
Ssc.6058.1.S1_at	*DUSP1*	similar to dual specificity protein phosphatase 1
Ssc.4871.1.S1_at	*CXCL2*	Macrophage inflammatory protein 2-alpha (CXCL2)
Ssc.17286.1.A1_at	*BTG2*	similar to B-cell translocation gene 2
Ssc.9707.1.A1_at	*BTG2*	similar to B-cell translocation gene 2
Ssc.4104.1.S1_at	*DDIT4*	similar to DNA-damage-inducible transcript 4 protein
Ssc.11269.1.A1_at	*IER2*	similar to immediate early response gene 2 protein
Ssc.8436.1.S1_at	*OSBP2*	similar to oxysterol-binding protein 2
Ssc.25461.1.S1_at	---	unknown
Ssc.2841.1.S1_at	*CREM*	similar to cAMP-responsive element modulator
Ssc.8031.1.A1_at	---	unknown
Ssc.7370.1.A1_at	---	unknown
Ssc.10822.1.S1_at	*EEF1G*	Elongation factor 1-gamma
Ssc.12095.1.S1_at	*CCD38*	similar to coiled-coil domain-containing protein 38
Ssc.5145.1.S1_a_at	*HSPA1B*	Heat shock 70 kDa protein 1 (HSP72)
Ssc.5145.2.S1_at	*HSPA1B*	Heat shock 70 kDa protein 1 (HSP72)
Ssc.5145.1.S1_at	*HSPA1B*	Heat shock 70 kDa protein 1 (HSP72)
Ssc.6163.2.S1_at	*ETS2*	similar to v-ets erythroblastosis virus E26 oncogene homolog 2
Ssc.2392.1.A1_at	*CCNL1*	similar to cyclin L1
Ssc.12365.1.A1_at	*ADAMTS1*	similar to A disintegrin and metalloproteinase with thrombospondin motifs 1 (ADAMTS1)
Ssc.1674.1.A1_at	*SLC2A3*	GLUT3
Ssc.16466.1.A1_at	*ATF3*	similar to activating transcription factor 3
Ssc.9473.1.A1_at	*BAG3*	similar to BAG family molecular chaperone regulator 3
Ssc.3108.1.A1_at	*PPP1R15A*	similar to protein phosphatase 1, regulatory (Inhibitor) subunit 15A
Ssc.9062.1.A1_at	*FOSB*	similar to FOSB
Ssc.1555.1.A1_at	*FOS*	similar to c-fos
Ssc.5547.1.A1_at	*FOSL2*	similar to FOS-like antigen 2
Ssc.21987.2.S1_at	*IFRD1*	Interferon-related developmental regulator 1
Ssc.3502.1.S1_at	*HSP40*	Heat shock 40 kDa protein 1
Ssc.10226.1.A2_at	*RHOB*	Rho-related GTP-binding protein RhoB
Ssc.10226.1.A1_at	*RHOB*	Rho-related GTP-binding protein RhoB

Cluster 7

probe set		gene annotation
Ssc.27072.1.S1_at	*CNOT3*	similar to CCR4-NOT transcription complex subunit 3
Ssc.12446.1.A1_at	*CASP4*	similar to caspase 4
Ssc.13622.1.S1_at	*AQP4*	similar to aquaporin 4
Ssc.31206.3.S1_at	*CGGBP1*	similar to CGG triplet repeat-binding protein 1
Ssc.5978.2.S1_a_at	*RHBDD2*	similar to rhomboid domain-containing protein 2
Ssc.31206.2.A1_at	*CGGBP1*	similar to CGG triplet repeat-binding protein 1
Ssc.22357.1.A1_at	*XPO1*	similar to exportin1
Ssc.6622.1.S1_at	*HPCAL1*	similar to hippocalcin-like 1
Ssc.24112.1.A1_at	*SEPT11*	similar to septin 11
Ssc.22438.1.A1_at	---	unknown
Ssc.3659.1.S1_at	*MLL*	similar to myeloid/lymphoid or mixed-lineage leukemia
Ssc.10056.1.A1_at	---	unknown
Ssc.22082.1.A1_at	*DAB1*	similar to disabled homolog 1
Ssc.21851.1.S1_at	---	unknown
Ssc.7179.3.S1_at	---	unknown
Ssc.16114.1.S1_at	*CACNA2D1*	Voltage-dependent calcium channel subunit alpha-2/delta-1
Ssc.23516.3.S1_at	*SATB1*	similar to DNA-binding protein SATB1
Ssc.30546.1.A1_at	---	unknown
Ssc.6410.3.S1_at	*VGLL4*	similar to transcription cofactor vestigial-like protein 4
Ssc.24693.2.S1_at	*GCLM*	similar to glutamate-cysteine ligase regulatory subunit
Ssc.14047.1.A1_at	---	unknown
Ssc.27352.1.S1_at	---	unknown
Ssc.23222.1.S1_at	*PDXP*	similar to pyridoxal phosphate phosphatase
Ssc.30674.1.S1_at	*LPPR3*	similar to lipid phosphate phosphatase-related protein type 3
Ssc.25483.1.S1_at	*CAMK4*	similar to calcium/calmodulin-dependent protein kinase type IV
Ssc.4253.1.S1_at	*TGFBR3*	TGF-beta receptor type III
Ssc.18641.1.A1_at	*PTPRT*	similar to receptor-type tyrosine-protein phosphatase T
Ssc.13793.2.S1_at	*RAD51AP1*	similar to RAD51-associated protein 1
Ssc.6249.2.S1_at	*MHC class1*	MHC class1
Ssc.24429.1.A1_at	*SSFA2*	similar to sperm-specific antigen 2
Ssc.30862.1.S1_at	*DNAJB9*	similar to DnaJ (Hsp40) homolog subfamily B member 9
Ssc.6906.1.A1_at	*FNDC3A*	similar to fibronectin type-III domain-containing protein 3a
Ssc.24179.1.A1_at	*ARF2*	similar to ADP-ribosylation factor 2
Ssc.7947.1.A1_at	*PREPL*	similar to prolyl endopeptidase-like
Ssc.25165.1.A1_at	---	similar to G protein beta1/gamma2 subunit-interacting factor 3
Ssc.25150.1.S1_at	---	unknown
Ssc.24428.1.A1_at	*DNM1L*	similar to dynamin-1-like protein
Ssc.25082.1.S1_at	*KIAA1128*	similar to protein GCAP14 homolog
Ssc.7182.1.A1_at	*BMPR2*	similar to bone morphogenetic protein receptor type-2
Ssc.7378.1.A1_at	*ATP6V1B2*	similar to vacuolar ATP synthase subunit B
Ssc.12486.1.S1_at	*CYP17A1*	Cytochrome P450 17A1
Ssc.18591.2.S1_at	*C20orf12*	similar to ankyrin repeat-containing protein C20orf12
Ssc.15912.1.S1_at	*CX43*	Connexin 43
Ssc.118.1.S1_at	*ATP6V1a1*	Vacuolar ATP synthase catalytic subunit A
Ssc.11382.1.S1_at	*SERINC1*	similar to serine incorporator 1
Ssc.27599.1.S1_at	*GLRB*	Glycine receptor subunit beta
Ssc.21606.1.S1_at	*GMFB*	similar to glia maturation factor beta
Ssc.246.1.S1_at	*ATP1B1*	Sodium/potassium-transporting ATPase subunit beta-1
Ssc.1094.2.S1_at	*GLO1*	similar to glyoxalase I
Ssc.6130.1.S1_at	*FBXW11*	similar to F-box/WD repeat protein 11
Ssc.26804.1.S1_at	*UBE2W*	similar to ubiquitin-conjugating enzyme E2 W
Ssc.23154.2.S1_at	*MKI67IP*	similar to MKI67 FHA domain-interacting nucleolar phosphoprotein
Ssc.13587.2.S1_at	*ANK3*	similar to ankyrin3
Ssc.12191.1.A1_at	*HSP90a*	Heat shock protein HSP 90-alpha
Ssc.17955.1.A1_at	*AZIN1*	similar to ornithine decarboxylase antizyme inhibitor
Ssc.19298.2.S1_at	*DHCR24*	similar to 24-dehydrocholesterol reductase
Ssc.11046.1.S1_at	*SEC22b*	similar to vesicle-trafficking protein SEC22b
Ssc.16028.1.S1_at	*TIMP3*	Metalloproteinase inhibitor 3
Ssc.30395.1.A1_at	---	unknown
Ssc.2691.1.A1_at	*ZDHHC17*	similar to palmitoyltransferase ZDHHC17
Ssc.22588.2.S1_at	*GABARAPL1*	similar to gamma-aminobutyric acid receptor-associated protein-like 1
Ssc.17592.1.S1_at	*ANKH*	similar to progressive ankylosis protein homolog
Ssc.18027.1.S1_at	*BTBD6*	similar to BTB/POZ domain-containing protein 6
Ssc.607.1.S1_at	*GLUT3*	GLUT3
Ssc.30016.3.A1_at	*TSC1*	Tuberous sclerosis 1 protein
Ssc.12493.1.A1_at	*PAFAH1B2*	similar to platelet-activating factor acetylhydrolase IB subunit beta
Ssc.26753.1.A1_at	*CACNA2D1*	similar to calcium channel, voltage-dependent, alpha 2/delta subunit 1
Ssc.26934.1.S1_at	*ETNK1*	similar to ethanolamine kinase 1
Ssc.17427.1.S1_at	*BASP1*	similar to brain acid soluble protein 1
Ssc.11756.3.A1_at	*CDC2L6*	similar to cell division cycle 2-like 6
Ssc.1081.3.A1_at	*MBNL1*	similar to muscleblind-like protein 1
Ssc.10003.1.S1_at	*TMEM30A*	similar to transmembrane protein 30A
Ssc.28306.1.A1_at	---	unknown
Ssc.10473.1.A1_at	*ZFP91*	similar to Zinc finger protein 91 homolog
Ssc.29002.1.A1_at	*GUCY1B3*	Guanylate cyclase soluble subunit beta-1
Ssc.30424.1.A1_at	*ZNF335*	similar to zinc finger protein 335
Ssc.1081.2.S1_at	*MBNL1*	similar to muscleblind-like protein 1
Ssc.8896.1.A1_at	---	unknown

Ssc.11035.1.S1_at	*KLHDC2*	similar to Kelch domain-containing protein 2
Ssc.12862.1.A1_at	*MAP1B*	similar to microtubule associated protein 1b
Ssc.22678.1.S1_at	---	unknown
Ssc.12959.1.A1_at	---	unknown
Ssc.18307.1.A1_at	*RBM25*	similar to probable RNA-binding protein 25
Ssc.20672.1.S1_at	*RNF103*	similar to ring finger protein 103
Ssc.1966.3.S1_at	*STX5*	similar to syntaxin 5
Ssc.24046.1.S1_at	*ANKRD50*	similar to ankyrin repeat domain-containing protein 50
Ssc.27501.1.S1_at	*GEMIN5*	similar to gem-associated protein 5
Ssc.8320.1.A1_at	*PREX1*	similar to phosphatidylinositol 3,4,5-trisphosphate-dependent RAC exchanger 1
Ssc.3802.1.S1_at	*NAP1L1*	similar to nucleosome assembly protein 1-like 1
Ssc.18335.2.A1_at	*PCNP*	similar to PEST proteolytic signal-containing nuclear protein
Ssc.11310.1.A1_at	*CHRDL1*	similar to chordin-like protein 1
Ssc.25021.1.S1_at	---	unknown
Ssc.26659.1.A1_at	*TFAP2A*	similar to transcription factor AP-2 alpha
Ssc.15984.2.A1_at	*GLUT4*	GLUT4
Ssc.18880.1.A1_at	---	similar to calcium/calmodulin-dependent serine protein kinase
Ssc.17566.1.S1_at	*DDB1*	similar to DNA damage-binding protein 1
Ssc.11158.3.S1_at	*PTP4A2*	similar to protein tyrosine phosphatase type IVA protein 2
Ssc.9182.1.A1_at	---	unknown
Ssc.22105.1.S1_at	*COX15*	similar to cytochrome c oxidase assembly protein COX15 homolog
Ssc.2302.1.S1_at	*SLC30A3*	similar to solute carrier family 30 member 3
Ssc.21905.1.S1_at	*SLC37A4*	similar to glucose-6-phosphate translocase
Ssc.26046.1.S1_at	*RBPJ*	similar to recombining binding protein suppressor of hairless
Ssc.8613.1.A1_at	*ARL15*	similar to ADP-ribosylation factor-like 15
Ssc.18464.1.S1_at	---	unknown
Ssc.16213.1.A1_x_at	*SLA-DRB2-2D*	MHC class II SLA-DRB2-2D
Ssc.17959.1.A1_at	*SATB1*	similar to DNA-binding protein SATB1
Ssc.27304.3.S1_s_at	*TMSB4Y*	Thymosin beta-4, Y-chromosomal
Ssc.4127.1.A1_at	*RND3*	similar to Rho-related GTP-binding protein RhoE
Ssc.23465.2.S1_at	*TJP2*	similar to tight junction protein 2
Ssc.8091.1.S1_at	*PREPL*	similar to prolyl endopeptidase-like
Ssc.22488.1.S1_at	---	unknown
Ssc.13853.1.A1_at	---	unknown
Ssc.3216.2.A1_at	*HNRPH2*	similar to heterogeneous nuclear ribonucleoprotein H
Ssc.6829.1.A1_at	---	unknown
Ssc.19298.1.A1_at	*DHCR24*	similar to 24-dehydrocholesterol reductase
Ssc.23545.2.A1_at	*PIP5K1A*	similar to phosphatidylinositol-4-phosphate-5 kinase, type 1 alpha
Ssc.18255.1.S1_at	*GGT4*	similar to gamma-glutamyltransferase 4
Ssc.21182.1.S1_at	*PFDN6*	similar to prefoldin subunit 6
Ssc.18635.1.S1_at	*SIVA1*	similar to apoptosis regulatory protein Siva
Ssc.1042.1.A1_at	*CNOT7*	similar to CCR4-NOT transcription complex, subunit 7
Ssc.17631.1.S1_at	*ZBTB47*	similar to zinc finger and BTB domain-containing protein 47
Ssc.11675.2.A1_at	*RC3H2*	similar to ring finger and CCCH-type zinc finger domain-containing protein 2
Ssc.23508.1.S1_at	*ERGIC1*	similar to endoplasmic reticulum-Golgi intermediate compartment protein 1
Ssc.17405.2.A1_at	*OAZ2*	similar to ornithine decarboxylase antizyme 2
Ssc.14178.1.A1_at	*GGPS1*	similar to geranylgeranyl pyrophosphate synthetase
Ssc.19334.1.S1_at	*GRB2*	similar to growth factor receptor-bound protein 2.
Ssc.28957.1.S1_at	*TNRC4*	similar to trinucleotide repeat-containing gene 4 protein
Ssc.22588.1.S1_at	*GABARAPL1*	similar to gamma-aminobutyric acid receptor-associated protein-like 1
Ssc.4235.1.S1_at	*PMSD4*	26S proteasome non-ATPase regulatory subunit 4
Ssc.16633.1.S1_at	*TROP*	Trophinin
Ssc.23767.1.S1_at	*POLR1E*	similar to DNA-directed RNA polymerase I subunit RPA49
Ssc.10983.2.S1_at	*PICALM*	similar to phosphatidylinositol-binding clathrin assembly protein
Ssc.28297.1.A1_at	---	unknown
Ssc.3195.1.S1_at	*TNRC15*	similar to trinucleotide repeat-containing protein 15
Ssc.27042.1.A1_at	---	unknown
Ssc.13862.2.S1_a_at	*TMEM131*	similar to transmembrane protein 131
Ssc.9066.1.S1_a_at	*HDAC3*	similar to histone deacetylase 3
Ssc.12078.2.S1_at	*SRP72*	similar to signal recognition particle 72 kDa
Ssc.24080.1.A1_at	*INA*	similar to alpha-internexin
Ssc.7628.1.A1_at	*CSDE1*	similar to cold shock domain-containing protein E1
Ssc.2172.1.S1_at	*NDRG2*	N-myc downstream regulated gene 2
Ssc.11673.1.S1_at	*UBE2E3*	similar to ubiquitin-conjugating enzyme E2 E3
Ssc.8434.1.A1_at	---	unknown
Ssc.5042.1.S1_at	*CCDC109A*	similar to coiled-coil domain-containing protein 109A
Ssc.24422.1.S1_at	*UBE2S*	similar to ubiquitin-conjugating enzyme E2 S
Ssc.11550.1.A1_at	*FBXW2*	similar to F-box/WD repeat-containing protein 2
Ssc.19417.1.S1_at	*DNAJC7*	similar to DnaJ (Hsp40) homolog subfamily C member 7
Ssc.12623.1.A1_at	*GGA3*	similar to golgi-localized, gamma ear-containing, ARF-binding protein 3
Ssc.12651.1.A1_at	*DIO2*	similar to type II iodothyronine deiodinase
Ssc.22809.1.S1_at	---	unknown
Ssc.9059.1.A1_at	*UBC*	Ubiquitin C
Ssc.12091.2.A1_at	*SMARCA1*	similar to probable global transcription activator SNF2L1
Ssc.18127.1.A1_at	*NIPA1*	similar to non-imprinted in Prader-Willi/Angelman syndrome region protein 1
Ssc.9406.2.S1_at	*LYPLA1*	similar to lysophospholipase 1
Ssc.20711.1.S1_at	*THBD*	similar to thrombomodulin
Ssc.10245.2.A1_a_at	*DCN*	Decorin
Ssc.8359.1.A1_at	*SEMA6A*	similar to semaphorin-6A

Ssc.4787.1.A1_at	*CCNL2*	similar to cyclin-L2
Ssc.29767.1.A1_at	*EXOC5*	similar to exocyst complex component 5
Ssc.30540.1.A1_at	*CBL*	E3 ubiquitin-protein ligase CBL
Ssc.1472.3.A1_at	---	unknown
Ssc.27378.1.S1_at	*MMS19*	similar to yeast nucleotide excision repair MMS19 homolog
Ssc.27811.3.A1_at	---	unknown
Ssc.938.1.S1_at	*TWSG1*	similar to twisted gastrulation protein homolog 1
Ssc.6911.1.A1_at	*ELAVL4*	similar to ELAV (Embryonic lethal, abnormal vision, Drosophila)-like 4
Ssc.1655.2.A1_at	*SLMAP*	similar to sarcolemmal membrane-associated protein
Ssc.30772.1.S1_at	*BAT1*	Spliceosome RNA helicase BAT1
Ssc.7810.1.A1_at	---	unknown
Ssc.1779.1.S1_at	*TBC1D10A*	similar to TBC1 domain family member 10A
Ssc.24827.1.A1_at	*CTAGE5*	similar to cutaneous T-cell lymphoma-associated antigen 5
Ssc.6737.2.A1_at	*ITGAV*	similar to integrin alpha-V
Ssc.11450.1.S1_at	*CLIC1*	chloride intracellular channel 1
Ssc.19413.1.A1_at	*SOX4*	similar to transcription factor SOX-4
Ssc.6093.3.A1_at	*MAP1B*	similar to microtubule associated protein 1b
Ssc.997.3.A1_at	*KDELR2*	similar to ER lumen protein retaining receptor 2
Ssc.14592.2.A1_at	---	unknown
Ssc.25855.1.S1_at	---	unknown
Ssc.8761.1.A1_at	*WSB2*	similar to WD repeat and SOCS box-containing 2
Ssc.11440.3.A1_at	---	unknown
Ssc.8774.2.A1_at	*SC4MOL*	C-4 methylsterol oxidase
Ssc.7633.2.A1_at	*ERBB2IP*	similar to Erbb2-interacting protein
Ssc.2780.1.A1_at	*METT11D1*	similar to methyltransferase 11 domain containing 1
Ssc.26420.1.A1_at	*TLK1*	similar to serine/threonine-protein kinase tousled-like 1
Ssc.3550.1.S1_at	*TM4SF1*	similar to transmembrane 4 L6 family member 1
Ssc.1355.1.S1_at	---	unknown
Ssc.8865.2.S1_at	*DUSP6*	similar to dual specificity protein phosphatase 6
Ssc.20607.2.A1_at	---	unknown
Ssc.18168.1.A1_at	*NT*	similar to neurotrimin
Ssc.5941.1.S1_at	*ACTN1*	similar to alpha-actinin-1
Ssc.6553.1.A1_at	*VPS16*	similar to vacuolar protein sorting-associated protein 16 homolog
Ssc.829.1.A1_at	---	unknown
Ssc.15900.1.S1_at	*CARTPT*	Cocaine- and amphetamine-regulated transcript protein
Ssc.26339.1.S1_a_at	*GNRHR2*	Receptor for gonadotropin releasing hormone II (GnRH II)
Ssc.8323.1.A1_at	---	unknown
Ssc.23994.1.A1_at	---	unknown
Ssc.7351.1.S1_at	*BTBD3*	similar to BTB/POZ domain-containing protein 3
Ssc.9984.1.A1_at	*KLF4*	Krueppel-like factor 4
Ssc.10881.1.S1_at	*JUNB*	JUNB
Ssc.8807.1.A1_at	*NR4A3*	similar to orphan nuclear receptor NR4A3
Ssc.24859.1.A1_at	*YWHAZ*	similar to tyrosine 3-monooxygenase/tryptophan 5-monooxygenase activation protein, zeta
Ssc.19946.1.S1_at	*Ig-lambda*	similar to Ig-lambda
Ssc.3771.1.A1_at	*ENAH*	similar to protein enabled homolog
Ssc.8671.1.A1_at	*PI15*	similar to peptidase inhibitor 15
Ssc.17100.1.S1_at	*S100A8*	similar to S100 calcium-binding protein A8
Ssc.26409.2.S1_at	*WNK1*	similar to serine/threonine-protein kinase WNK1

Cluster 8

probe set		gene annotation
Ssc.6736.1.S1_at	*MSX2*	similar to homeobox protein MSX-2.
Ssc.26638.1.S1_at	*TMEM56*	similar to transmembrane protein 56
Ssc.15492.1.S1_at	*TRIB1*	similar to tribbles homolog 1
Ssc.11197.1.S1_at	*HSP27*	Heat shock protein beta-1 (HSP27)
Ssc.9380.2.S1_a_at	*GADD45B*	similar to growth arrest and DNA-damage-inducible protein GADD45 beta
Ssc.2033.1.S1_at	*CRY1*	similar to cryptochrome-1
Ssc.18076.1.A1_at	*GSN*	Gelsolin
Ssc.28567.1.A1_at	*PGM2*	similar to phosphoglucomutase-2
Ssc.15439.1.A1_at	*FUCA2*	similar to plasma alpha-L-fucosidase
Ssc.9108.1.S1_at	---	unknown
Ssc.2464.1.S1_at	*STC1*	similar to stanniocalcin-1
Ssc.21300.1.S1_at	---	unknown
Ssc.7093.1.A1_at	*HPSE*	similar to heparanase
Ssc.6356.1.S1_at	*ODC*	Ornithine decarboxylase
Ssc.26274.1.S1_at	*PIM3*	similar to serine/threonine-protein kinase Pim-3
Ssc.18577.1.S1_at	*DYRK3*	similar to dual specificity tyrosine-phosphorylation-regulated kinase 3
Ssc.18577.2.A1_at	*DYRK3*	similar to dual specificity tyrosine-phosphorylation-regulated kinase 3
Ssc.12026.1.A1_at	*CASP7*	similar to caspase-7
Ssc.22196.1.A1_at	*VKORC1L1*	similar to vitamin K epoxide reductase complex subunit 1-like protein 1
Ssc.21122.1.S1_at	*STK10*	similar to serine/threonine-protein kinase 10
Ssc.30617.1.S1_at	---	unknown
Ssc.944.1.A1_at	*LMNA*	similar to lamin-A/C
Ssc.8706.1.S1_at	*RGS3*	similar to regulator of G-protein signaling 3
Ssc.6382.1.A1_at	*PPP1R3B*	similar to protein phosphatase 1 regulatory subunit 3B
Ssc.30147.1.A1_at	---	unknown
Ssc.10786.1.A1_at	*SIRT1*	similar to NAD-dependent deacetylase sirtuin-1
Ssc.11170.1.S1_at	*PDXK*	Pyridoxal kinase
Ssc.4227.1.S1_at	*CLDN5*	Claudin 5

Ssc.30658.2.S1_at	*FBXL14*	similar to F-box and leucine-rich repeat protein 14
Ssc.10237.1.S1_at	*HSPB8*	Heat shock protein beta-8 (HSP22)
Ssc.18038.1.A1_at	*MAP3K8*	similar to mitogen-activated protein kinase kinase kinase 8
Ssc.19389.1.A1_at	*NMES1*	similar to normal mucosa of esophagus-specific gene 1 protein
Ssc.9586.1.S1_at	*SDPR*	similar to serum deprivation-response protein
Ssc.22477.1.S1_at	*SIX4*	similar to sine oculis homeobox homolog 4
Ssc.18072.1.A1_at	*OSMR*	similar to oncostatin-M receptor
Ssc.10025.3.S1_at	*CEBPD*	similar to CCAAT/enhancer-binding protein delta
Ssc.13699.1.A1_at	---	unknown
Ssc.18240.2.A1_at	*MOAP1*	similar to modulator of apoptosis 1
Ssc.7116.1.A1_at	*NT5C3*	similar to cytosolic 5-nucleotidase 3
Ssc.15740.1.S2_at	*VEGFA*	Vascular endothelial growth factor A
Ssc.31172.1.S1_at	*SDC1*	similar to syndecan-1
Ssc.19350.1.S1_at	*IER5*	similar to immediate early response gene 5 protein
Ssc.17243.1.S1_at	*DNAJA4*	DnaJ (Hsp40) homolog, subfamily A, member 4
Ssc.11784.1.S1_at	*TIMP1*	Metalloproteinase inhibitor 1
Ssc.196.1.S1_at	*PLAT*	Tissue-type plasminogen activator
Ssc.20386.1.S1_at	*CDKN2AIP*	similar to CDKN2A interacting protein
Ssc.26179.1.S1_at	*MIDN*	similar to midnolin
Ssc.16605.1.S1_at	*DDIT3*	similar to DNA damage-inducible transcript 3
Ssc.8528.1.A1_at	---	unknown
Ssc.17312.1.A1_at	---	unknown
Ssc.9781.1.S1_at	*SERPINE1*	Plasminogen activator inhibitor 1
Ssc.13649.1.S1_at	*BAG3*	similar to BAG family molecular chaperone regulator 3
Ssc.14117.1.A1_at	*DEPDC7*	DEP domain containing 7
Ssc.20913.1.S1_at	*GADD45A*	Growth arrest and DNA-damage-inducible protein GADD45 alpha
Ssc.3139.1.A1_at	*RGS2*	Regulator of G-protein signaling 2
Ssc.3909.1.A1_at	*SLC16A9*	similar to solute carrier family 16 member 9
Ssc.9380.1.S1_at	*GADD45B*	similar to growth arrest and DNA-damage-inducible protein GADD45 beta
Ssc.22952.1.S1_at	*TMEM128*	similar to transmembrane protein 128
Ssc.114.1.S1_at	*HSP70*	Heat shock 70 kDa protein 6 (HSP70B)
Ssc.639.1.A1_at	*GEM*	GTP-binding protein GEM
Ssc.10327.1.A1_at	---	unknown
Ssc.16882.1.A1_at	*NFIL3*	similar to nuclear factor, interLeukin 3 regulated
Ssc.9372.1.A1_at	*NFKBIZ*	similar to NF-kappa-B inhibitor zeta
Ssc.26230.1.S1_at	*RRAD*	similar to GTP-binding protein RAD, RAS associated with diabetes
Ssc.16392.2.A1_at	*MKNK2*	similar to MAP kinase-interacting serine/threonine-protein kinase 2
Ssc.20585.1.S1_at	*GADD45G*	similar to Growth arrest and DNA-damage-inducible protein GADD45 gamma
Ssc.14764.1.A1_at	*GADD45B*	similar to growth arrest and DNA-damage-inducible protein GADD45 beta
Ssc.8811.1.S1_at	---	unknown
Ssc.24938.1.S1_at	*METRNL*	similar to meteorin-like protein
Ssc.19629.2.S1_s_at	*EGR1*	similar to early growth response protein 1
Ssc.17522.1.A1_at	*ALDH1A2*	similar to aldehyde dehydrogenase family 1 member A2
Ssc.14281.3.A1_at	---	unknown
Ssc.21942.3.A1_a_at	*TSKU*	similar to Tsukushin
Ssc.5239.1.S1_at	---	unknown
Ssc.295.1.A1_at	*GLS*	Glutaminase
Ssc.9586.2.S1_at	*SDPR*	similar to serum deprivation-response protein
Ssc.5082.1.A1_at	---	unknown
Ssc.16434.1.A1_at	*NOV*	similar to nephroblastoma overexpressed gene protein homolog
Ssc.888.1.A1_at	*ERRFI1*	similar to ERBB receptor feedback inhibitor 1
Ssc.5737.1.S1_at	*CDKN1A*	similar to cyclin-dependent kinase inhibitor 1 (p21)
Ssc.924.2.A1_at	*THBS1*	Thrombospondin-1
Ssc.28305.1.A1_at	*TACC1*	similar to transforming acidic coiled-coil-containing protein 1
Ssc.115.1.S1_s_at	*HMOX1*	Heme oxygenase 1
Ssc.16451.1.A1_at	*ADAMTS1*	similar to A disintegrin and metalloproteinase with thrombospondin motifs 1 (ADAMTS1)
Ssc.8843.1.A1_at	---	unknown
Ssc.22550.1.A1_at	*C-JUN*	C-JUN
Ssc.8528.2.S1_at	---	unknown
Ssc.6564.1.S1_at	*RASL11A*	similar to RAS-like protein 11A
Ssc.16814.2.S1_at	*C19orf61*	similar to uncharacterized protein C19orf61.
Ssc.9075.1.A1_at	*C-JUN*	C-JUN
Ssc.5070.1.A1_at	*CCNG1*	Cyclin G1
Ssc.27556.1.S1_at	*FAM46C*	similar to protein FAM46C
Ssc.8766.1.A1_at	*CENPE*	similar to centromeric protein E
Ssc.6323.1.S1_at	*ADFP*	Adipophilin
Ssc.3509.1.S1_at	*HK2*	similar to hexokinase 2
Ssc.15664.1.S1_at	---	unknown
Ssc.19558.1.S1_at	*CD2BP2*	similar to CD2 antigen cytoplasmic tail-binding protein 2
Ssc.15598.1.S1_at	*INSIG1*	Insulin-induced gene 1 protein
Ssc.11187.1.S1_at	*ICAM-1*	Intercellular adhesion molecule 1

Cluster 9<br>probe set		gene annotation
Ssc.3251.1.A1_at	*PPARD*	Peroxisome proliferator-activated receptor delta
Ssc.5051.1.S1_at	---	unknown
Ssc.10968.1.S1_at	---	unknown
