# Supplementary material for: Effect of methylene blue on the genomic response to reperfusion injury induced by cardiac arrest and cardiopulmonary resuscitation in porcine brain
Source: BMC Med Genomics. 2010 Jul 1;3:27. doi: 10.1186/1755-8794-3-27 (PMC2904268; doi:10.1186/1755-8794-3-27)
Supplement: Additional file 2 — Processes and Proteins affected by ROSC and MB respectively. table. [file 1755-8794-3-27-S2.PDF]

Additional file 2
Processes and Proteins affected by ROSC and MB respectively

FC = Fold change
Indicates significant up-regulation by Rosc after gene filtering
Indicates significant down-regulation by Rosc after gene filtering

Indicates significant up-regulation by MB after gene filtering
Indicates significant down-regulation by MB after gene filtering

biological process / function	probe set	gene symbol	FC-Rosc30	FC-Rosc60	FC-Rosc180	FC-MB30	FC-MB60	FC-MB180	gene name
Inflammation									
cell adhesion	Ssc.11187.1.S1_at	***ICAM-1***	1,4	1,5	1,7	-1,5	-1,3	-1,1	Intercellular adhesion molecule 1
chemokine	Ssc.4871.1.S1_at	***CXCL2***	19,1	3,8	1,4	1,0	1,2	2,5	Macrophage inflammatory protein 2-alpha (CXCL2)
matrix degrading enzyme	Ssc.7093.1.A1_at	***HPSE***	2,0	2,5	3,2	-1,5	-1,4	-1,2	similar to heparanase
reponse to cytokine stimulus	Ssc.18072.1.A1_at	***OSMR***	-1,5	-1,1	3,7	1,8	1,3	1,3	similar to oncostatin-M receptor
Apoptosis/programmed cell death									
caspase-dependent response	Ssc.12026.1.A1_at	***CASP7***	1,0	-1,1	1,8	-1,5	-1,1	1,0	similar to caspase-7
caspase-dependent response	Ssc.944.1.A1_at	***LMNA***	1,0	1,0	1,7	-1,1	1,1	-1,1	similar to lamin-A/C
caspase-dependent response	Ssc.6163.2.S1_at	***ETS2***	1,5	1,5	1,4	1,0	1,0	1,2	similar to v-ets erythroblastosis virus E26 oncogene homolog 2
caspase-dependent response	Ssc.18240.2.A1_at	***MOAP1***	1,2	1,3	2,3	1,3	1,4	1,1	similar to modulator of apoptosis 1
caspase-dependent response	Ssc.24864.1.A1_at	***MEIS1***	1,5	1,4	-1,1	1,4	-1,5	1,0	similar to homeobox protein Meis1
p53 and caspase-dependent response	Ssc.5737.1.S1_at	***CDKN1A***	1,5	1,9	2,0	1,2	1,2	1,1	similar to cyclin-dependent kinase inhibitor 1 (p21)
p53-dependent response	Ssc.11269.1.A1_at	***IER2***	2,8	2,0	2,2	-1,1	1,1	-1,1	similar to immediate early response gene 2 protein
p53-dependent response	Ssc.5070.1.A1_at	***CCNG1***	1,7	1,5	1,6	-1,4	-1,1	1,1	Cyclin G1
p53-dependent response	Ssc.2464.1.S1_at	***STC1***	1,5	1,7	2,3	-1,2	-1,3	-1,2	similar to stanniocalcin-1
p53-dependent response	Ssc.20386.1.S1_at	***CDKN2AIP/CARF***	1,2	1,2	1,8	1,2	1,2	1,2	similar to CDKN2A interacting protein
p53-dependent response	Ssc.21987.1.A1_at	***IFRD1***	1,2	1,4	1,5	-1,5	-1,8	-1,6	Interferon-related developmental regulator 1
P53 / MAPK pathway	Ssc.20913.1.S1_at	***GADD45A***	3,1	3,3	4,4	1,1	1,1	1,2	Growth arrest and DNA-damage-inducible protein GADD45 alpha
P53 / MAPK pathway	Ssc.9380.1.S1_at	***GADD45B***	2,2	2,2	3,4	1,0	1,1	1,1	similar to growth arrest and DNA-damage-inducible protein GADD45 beta
P53 / MAPK pathway	Ssc.20585.1.S1_at	***GADD45G***	1,2	1,6	2,9	1,3	1,1	-1,1	similar to Growth arrest and DNA-damage-inducible protein GADD45 gamma
MAPK pathway	Ssc.888.1.A1_at	***ERRFI1***	1,2	1,6	1,9	1,3	1,1	1,1	similar to ERBB receptor feedback inhibitor 1
MAPK pathway	Ssc.18038.1.A1_at	***MAP3K8***	1,2	1,4	2,6	1,4	1,2	1,4	similar to mitogen-activated protein kinase kinase kinase 8
MAPK pathway	Ssc.16392.2.A1_at	***MKNK2***	1,1	1,3	1,6	1,0	1,0	1,0	similar to MAP kinase-interacting serine/threonine-protein kinase 2
MAPK pathway	Ssc.16039.1.S1_at	***RGS1***	5,7	2,5	1,6	1,1	1,1	1,3	Regulator of G-protein signaling 1
MAPK pathway	Ssc.3139.1.A1_at	***RGS2***	1,6	1,7	2,4	1,1	1,0	1,0	Regulator of G-protein signaling 2
MAPK pathway	Ssc.8706.1.S1_at	***RGS3***	1,1	1,0	1,9	-1,1	1,0	-1,2	similar to regulator of G-protein signaling 3
neuronal cell death / inflammation	Ssc.19629.2.S1_s_at	***EGR1***	1,3	1,6	1,9	1,8	1,1	1,1	similar to early growth response protein 1
neuronal cell death	Ssc.10226.1.A1_at	***RHOB***	3,5	2,7	1,1	-1,6	3,0	5,2	Rho-related GTP-binding protein RhoB
neuronal cell death	Ssc.2841.1.S1_at	***CREM***	1,7	2,1	1,5	-1,2	-1,4	1,0	similar to cAMP-responsive element modulator
modulator of apoptosis / inflammation	Ssc.12365.1.A1_at	***ADAMTS1***	6,1	8,4	8,6	-1,2	-1,4	-1,3	similar to A disintegrin and metalloproteinase with thrombospondin motifs 1 (ADAMTS1)
cell death / proteolysis	Ssc.196.1.S1_at	***t-PA***	1,1	1,3	1,8	1,3	1,1	1,2	Tissue-type plasminogen activator
neuronal survival	Ssc.23632.1.S1_at	***DYNC1I2***	-2,2	-2,7	-4,3	-1,1	1,1	1,3	similar to cytoplasmic dynein 1 intermediate chain 1/2
antiapoptotic (casp3 inhibitor)	Ssc.19298.2.S1_at	***DHCR24***	1,5	1,4	1,2	1,5	2,0	2,0	similar to 24-dehydrocholesterol reductase
antiapoptotic (casp3 inhibitor)	Ssc.23545.2.A1_at	***PIP5K1A***	-1,2	1,0	1,3	5,0	4,8	3,5	similar to phosphatidylinositol-4-phosphate-5 kinase, type 1 alpha
antiapoptotic	Ssc.6911.1.A1_at	***ELAVL4***	-1,1	1,3	1,4	3,0	1,4	2,0	similar to ELAV (Embryonic lethal, abnormal vision, Drosophila)-like 4
pro-survival p53 target	Ssc.4127.1.A1_at	***RND3***	-1,4	-1,1	1,1	3,2	2,3	1,7	similar to Rho-related GTP-binding protein RhoE
decreases p53, gadd45g and caspase3	Ssc.7628.1.A1_at	***CSDE1***	1,6	1,7	1,2	4,0	3,7	4,5	similar to cold shock domain-containing protein E1
anti-apoptotic	Ssc.17566.1.S1_at	***DDB1***	1,0	1,0	1,0	2,6	2,1	2,1	similar to DNA damage-binding protein 1
MAPK inhibitor	Ssc.8865.2.S1_at	***DUSP6***	-2,3	-1,1	-1,1	2,8	1,6	2,4	similar to dual specificity protein phosphatase 6
MAPK inhibitor	Ssc.24859.1.A1_at	***YWHAZ***	3,1	2,0	-1,3	2,3	1,8	4,0	'similar to tyrosine 3-monooxygenase/tryptophan 5-monooxygenase activation protein, zeta
p53 apoptotic target	Ssc.26993.1.S1_at	***SMARCA4***	-1,1	1,0	1,0	-1,8	-1,7	-1,6	similar to ATP-dependent helicase SMARCA4
caspase-dependent response	Ssc.17433.1.S1_at	***NEO1***	1,0	-1,1	-1,2	-1,6	-1,6	-1,4	similar to neogenin
autophagy	Ssc.2648.1.S1_at	***TMEM49***	1,1	-1,1	1,0	-2,2	-1,6	-1,5	similar to transmembrane protein 49
neuronal cell death	Ssc.6071.1.S1_at	***I2PP2A/SET***	1,0	1,0	1,1	-1,6	-1,6	-1,6	similar to phosphatase 2A inhibitor I2PP2A
mitochondrial apoptosis / pore complex	Ssc.16638.1.S1_at	***SLC25A6***	-1,2	-1,1	1,1	-1,2	-1,2	-1,7	ADP/ATP translocase 3
apoptosis marker	Ssc.5535.1.S1_at	***SAFB***	-1,1	-1,1	1,0	-1,6	-1,4	-1,6	similar to scaffold attachment factor B
ER stress response									
ER stress marker	Ssc.16605.1.S1_at	***DDIT3***	1,3	1,4	2,2	1,4	1,3	1,2	similar to DNA damage-inducible transcript 3

ER stress p53 activator	Ssc.16466.1.A1_at	***ATF3***	19,1	15,6	9,5	-1,2	1,3	1,0	similar to activating transcription factor 3
protein synthesis restoration	Ssc.3108.1.A1_at	***PPP1R15A/GADD34***	3,4	2,4	2,6	1,1	1,4	1,1	similar to protein phosphatase 1, regulatory (Inhibitor) subunit 15A
ER stress apoptotic p53 target	Ssc.4104.1.S1_at	***DDIT4***	2,0	1,2	1,5	-1,1	1,0	-1,1	similar to DNA-damage-inducible transcript 4 protein
ER stress inhibitor of translation	Ssc.7274.1.A1_at	***EIF2AK2***	1,7	1,5	1,1	-2,1	-1,2	-1,3	Eukaryotic translation initiation factor 2-alpha kinase 2
ERAD	Ssc.11308.1.A1_at	***EDEM3***	-1,1	-1,1	1,1	-1,2	-1,7	-1,4	similar to ER degradation-enhancing alpha-mannosidase-like 3
ER stress apoptosis inhibitor	Ssc.30862.1.S1_at	***DNAJB9***	1,5	1,5	1,1	1,9	1,9	2,5	similar to DnaJ (Hsp40) homolog subfamily B member 9
Endogenous defence mechanism									
AP-1 complex	Ssc.1555.1.A1_at	***FOS***	4,9	4,1	3,3	1,2	1,1	1,1	similar to c-fos
AP-1 complex	Ssc.9062.1.A1_at	***FOSB***	6,0	6,1	4,8	1,5	1,2	1,1	similar to FOSB
AP-1 complex	Ssc.5547.1.A1_at	***FOSL2***	2,0	2,2	2,0	1,1	1,2	1,0	similar to FOS-like antigen 2
AP-1 complex	Ssc.22550.1.A1_at	***C-JUN***	1,7	1,8	2,1	1,2	1,0	1,1	C-JUN
MAPK inhibitor / anti-inflammatory	Ssc.6058.1.S1_at	***DUSP1***	5,7	2,5	1,9	1,0	1,2	1,2	similar to dual specificity protein phosphatase 1
casp3 inhibitor	Ssc.18076.1.A1_at	***GSN***	1,2	1,3	1,8	1,0	-1,3	-1,1	Gelsolin
casp3 inhibitor	Ssc.6736.1.S1_at	***MSX2***	-1,4	1,5	1,7	1,8	-1,3	-1,1	similar to homeobox protein MSX-2.
anti-apoptotic	Ssc.9720.1.A1_at	***CYR61***	10,6	7,3	5,3	-1,3	-1,2	-1,1	similar to cysteine-rich, angiogenic inducer 61
anti-apoptotic	Ssc.26274.1.S1_at	***PIM3***	1,1	1,2	2,0	-1,1	1,0	-1,2	similar to serine/threonine-protein kinase Pim-3
anti-apoptotic / MMP inhibitor	Ssc.11784.1.S1_at	***TIMP1***	1,2	1,4	2,3	1,5	1,4	1,4	Metalloproteinase inhibitor 1
anti-apoptotic / tPA inhibitor	Ssc.9781.1.S1_at	***PAI-1/SERPINE1***	2,0	3,7	5,2	1,2	1,3	1,1	Plasminogen activator inhibitor 1
neuroprotective	Ssc.15740.1.S2_at	***VEGFA***	1,2	1,2	1,9	1,1	1,0	1,1	Vascular endothelial growth factor A
neuroprotective	Ssc.5737.1.S1_at	***CDKN1A/p21***	1,5	1,9	2,0	1,2	1,2	1,1	similar to cyclin-dependent kinase inhibitor 1 (p21)
neuroprotective	Ssc.10786.1.A1_at	***SIRT1***	-1,1	1,5	2,3	-1,7	-1,5	-1,2	similar to NAD-dependent deacetylase sirtuin-1
neuroprotective	Ssc.115.1.S1_s_at	***HMOX1***	3,0	2,7	3,8	1,5	1,9	1,4	Heme oxygenase 1
neuroprotective	Ssc.10776.1.A1_at	***HMOX2***	5,7	3,7	2,7	-1,3	-1,5	-1,5	similar to heme oxygenase 2
Heat shock proteins									
chaperone / antiapoptotic	Ssc.5145.1.S1_a_at	***HSP72***	11,9	11,0	12,8	-1,2	1,2	-1,1	Heat shock 70 kDa protein 1 (HSP72)
chaperone / antiapoptotic	Ssc.114.1.S1_at	***HSP70***	25,6	30,5	45,7	-1,5	1,1	1,1	Heat shock 70 kDa protein 6 (HSP70B)
MAPK pathway	Ssc.11197.1.S1_at	***HSP27***	1,8	1,5	2,3	-1,5	1,0	-1,1	Heat shock protein beta-1 (HSP27)
chaperone / neurite outgrowth	Ssc.3502.1.S1_at	***HSP40/DNAJB1***	5,6	5,9	4,0	1,0	1,6	1,4	Heat shock 40 kDa protein 1
chaperone	Ssc.17243.1.S1_at	***DNAJA4***	1,2	1,3	1,7	1,1	1,1	1,1	DnaJ (Hsp40) homolog, subfamily A, member 4
chaperone regulator / anti-apoptotic	Ssc.9473.1.A1_at	***BAG3***	31,9	22,7	11,7	-1,3	1,5	1,7	similar to BAG family molecular chaperone regulator 3
chaperone / HSP70 co-chaperone	Ssc.12191.1.A1_at	***HSP90***	2,4	2,2	1,1	1,8	3,3	4,8	Heat shock protein HSP 90-alpha
chaperone	Ssc.10237.1.S1_at	***HSP22***	1,0	1,2	2,3	1,4	1,2	1,4	Heat shock protein beta-8 (HSP22)
chaperone	Ssc.19417.1.S1_at	***DNAJC7***	1,1	1,0	-1,3	2,0		2,9	similar to DnaJ (Hsp40) homolog subfamily C member 7
HSP70 co-chaperone	Ssc.1180.1.S1_at	***DNAJB2***	1,0	-1,1	1,1	-1,9	-1,8	-1,6	similar to DnaJ (Hsp40) homolog subfamily B member 2
Neurogenesis									
neurogenesis regulation	Ssc.26179.1.S1_at	***MIDN***	1,1	1,3	2,0	1,6	1,3	1,2	similar to midnolin
p53-dependent response / antiapoptotic / neuronal differentiation	Ssc.17286.1.A1_at	***BTG2***	6,5	4,4	2,4	0,9	1,0	1,1	similar to B-cell translocation gene 2
motoneuron growth and survival	Ssc.16882.1.A1_at	***NFIL3***	2,3	2,8	3,6	-1,1	-1,2	1,1	similar to nuclear factor, interLeukin 3 regulated
glial cell differentiation / axonal extension	Ssc.24938.1.S1_at	***METRNL***	-1,2	1,2	1,3	1,6	1,1	1,0	similar to meteorin-like protein
axon regeneration	Ssc.31172.1.S1_at	***SDC1***	2,3	2,3	4,2	1,8	1,0	1,1	similar to syndecan-1
neurite outgrowth	Ssc.16466.1.A1_at	***ATF3***	19,1	15,6	9,5	-1,2	1,3	1,0	similar to activating transcription factor 3
postischemic neurogenesis activator	Ssc.15740.1.S2_at	***VEGFA***	1,2	1,2	1,9	1,1	1,0	1,1	Vascular endothelial growth factor A
microtubule-binding / chaperone-like	Ssc.22588.2.S1_at	***GABARAPL1***	1,4	1,3	-1,4	3,5	6,2	6,9	similar to gamma-aminobutyric acid receptor-associated protein-like 1
microtubule-dependent synaptic and neuron growth	Ssc.12862.1.A1_at	***MAP1B***	1,7	1,7	1,1	4,5	3,9	6,1	similar to microtubule associated protein 1b
vesicle targeting / neurite outgrowth	Ssc.29767.1.A1_at	***EXOC5***	-1,1	1,3	1,3	2,0	1,2	1,4	similar to exocyst complex component 5
synaptic growth and axonal microtubules regulator	Ssc.18127.1.A1_at	***NIPA1***	1,6	2,0	-1,1	1,6	1,3	2,7	similar to non-imprinted in Prader-Willi/Angelman syndrome region protein 1
cytoskeleton	Ssc.24080.1.A1_at	***INA***	1,7	1,7	1,2	2,7	3,1	6,6	similar to alpha-internexin
regulation of microtubule dynamics / adult neurogenesis	Ssc.25483.1.S1_at	***CAMK4***	-1,3	1,0	-1,1	2,6	1,7	1,6	similar to calcium/calmodulin-dependent protein kinase type IV
neuronal guidance/microtubule dynamics	Ssc.8359.1.A1_at	***SEMA6A***	-1,1	1,5	1,4	2,5	1,0	1,7	similar to semaphorin-6A
microtubule stability	Ssc.24112.1.A1_at	***SEPT11***	1,5	-1,3	-1,2	1,3	2,4	2,3	similar to septin 11
slow axonal transport microtubule-dependent motor	Ssc.10198.1.A1_at	***KIF5A***	1,1	1,0	-1,1	-1,6	-1,4	-1,2	similar to kinesin heavy chain isoform 5A
transport microtubule-dependent motor	Ssc.2401.1.A1_at	***KLC1***	1,1	1,1	1,0	-2,0	-2,1	-1,8	similar to kinesin light chain 1
neurite outgrowth	Ssc.17427.1.S1_at	***BASP1***	-1,1	1,0	-1,4	3,1	2,0	2,4	similar to brain acid soluble protein 1
neurite outgrowth	Ssc.15912.1.S1_at	***CX43***	1,8	3,5	3,1	9,0	7,9	8,9	Connexin 43
neurite outgrowth	Ssc.3550.1.S1_at	***TM4SF1***	-1,3	1,1	-1,2	2,1	1,2	1,9	similar to transmembrane 4 L6 family member 1
neurite outgrowth	Ssc.18168.1.A1_at	***NT***	-1,3	1,0	1,2	2,4	2,2	1,9	similar to neurotrimin
neurite outgrowth	Ssc.12091.2.A1_at	***SMARCA1***	1,3	1,6	1,5	2,0	1,4	1,9	similar to probable global transcription activator SNF2L1
pro-survival p53 target / neurite extension	Ssc.4127.1.A1_at	***RND3***	-1,4	-1,1	1,1	3,2	2,3	1,7	similar to Rho-related GTP-binding protein RhoE
pro-survival / axon regeneration	Ssc.27365.1.S1_at	***RUFY3***	1,2	-3,3	-3,3	1,2	7,0	1,9	similar to protein RUFY3

MAPK regulator / neuron and glia growth and differentiation factor	Ssc.21606.1.S1_at	***GMFB***	1,5	1,9	1,2	3,1	3,6	3,3	similar to glia maturation factor beta
axon regeneration	Ssc.8774.2.A1_at	***SC4MOL***	1,7	1,8	1,3	2,2	1,4	2,5	C-4 methylsterol oxidase
synaptogenesis	Ssc.18880.1.A1_at	***CASK***	1,9	2,0	1,9	3,1	2,2	3,7	similar to calcium/calmodulin-dependent serine protein kinase
neuronal migration	Ssc.12493.1.A1_at	***PAFAH1B2***	2,5	2,4	1,3	2,2	3,4	2,8	similar to platelet-activating factor acetylhydrolase IB subunit beta
neuronal migration	Ssc.22082.1.A1_at	***DAB1***	-1,3	-1,3	1,1	2,3	1,9	1,2	similar to disabled homolog 1
neuronal migration	Ssc.8320.1.A1_at	***PREX1***	1,9	1,6	-1,1	2,6	2,4	4,7	similar to phosphatidylinositol 3,4,5-trisphosphate-dependent RAC exchanger 1
neurogenesis repressor	Ssc.8174.1.A1_at	***OLIG2***	1,2	1,2	1,4	-1,8	-1,9	-1,4	similar to Oligodendrocyte transcription factor 2
apoptotic, axon guidance	Ssc.17433.1.S1_at	***NEO1***	1,0	-1,1	-1,2	-1,6	-1,6	-1,4	similar to neogenin
axon guidance	Ssc.23810.3.A1_at	***NFIA***	1,1	1,1	1,2	-1,6	-1,7	-1,6	similar to nuclear factor I/A
Neuroprotection									
gap junction	Ssc.15912.1.S1_at	***CX43***	1,8	3,5	3,1	9,0	7,9	8,9	Connexin 43
APC activator (anti-inflammatory, cytoprotective, neurogenetic)	Ssc.20711.1.S1_at	***THBD***	1,2	2,2	1,5	2,3	1,3	1,7	similar to thrombomodulin
water-specific channel	Ssc.13622.1.S1_at	***AQP4***	1,8	1,3	1,1	1,1	1,4	1,6	similar to aquaporin 4
neuronal development and survival	Ssc.15900.1.S1_at	***CART***	1,9	2,2	1,5	1,7	1,0	1,6	Cocaine- and amphetamine-regulated transcript protein
MAPK inhibitor	Ssc.8865.2.S1_at	***DUSP6***	-2,3	-1,1	-1,1	2,8	1,6	2,4	similar to dual specificity protein phosphatase 6
MAPK inhibitor	Ssc.24859.1.A1_at	***YWHAZ***	3,1	2,0	-1,3	2,3	1,8	4,0	'similar to tyrosine 3-monooxygenase/tryptophan 5-monooxygenase activation protein, zeta
Neurotransmitter release									
synaptic neurotransmission	Ssc.11706.2.A1_at	***DTNA***	1,0	-1,3	-2,1	-1,1	-1,1	1,9	similar to dystrobrevin alpha
SNARE / neurotransmitter release	Ssc.27354.1.S1_at	***STXBP5***	-1,8	-1,3	-9,6	3,0	3,3	1,4	similar to syntaxin-binding protein 5
SNARE / neurotransmitter release	Ssc.13474.1.S1_at	***SNAP25/SNSP25***	-1,9	-2,8	-2,9	1,2	1,1	-1,1	Similar to Synaptosomal-associated 25 kDa protein
exocytosis / neurotransmitter release	Ssc.12933.1.S1_at	***CADPS2***	-1,4	-1,2	-2,2	1,3	1,2	1,4	Similar to Calcium-dependent secretion activator 2
synaptic neurotransmission	Ssc.8492.1.A1_at	***RIMS2***	-1,4	-1,8	-2,5	1,0	1,3	1,0	Similar to Regulating synaptic membrane exocytosis protein 2
Vesicle transport									
retrograde transport	Ssc.23632.1.S1_at	***DYNC1I2***	-2,2	-2,7	-4,3	-1,1	1,1	1,3	similar to cytoplasmic dynein 1 intermediate chain 1/2
ER-vesicle transport	Ssc.11046.1.S1_at	***SEC22b***	2,0	1,8	1,4	1,6	2,5	2,6	similar to vesicle-trafficking protein SEC22b
ER/Golgi vesicle transport	Ssc.1966.3.S1_at	***STX5***	1,5	1,4	1,2	2,1	1,7	2,5	similar to syntaxin 5
ER/Golgi vesicle transport	Ssc.997.3.A1_at	***KDELR2***	1,2	1,4	-1,2	2,3	1,6	2,9	similar to ER lumen protein retaining receptor 2
ER/Golgi vesicle transport	Ssc.23508.1.S1_at	***ERGIC1***	1,0	1,0	1,0	2,0	2,3	2,4	similar to endoplasmic reticulum-Golgi intermediate compartment protein 1
vesicle-mediated protein transport to lysosome	Ssc.6553.1.A1_at	***VPS16***	-1,2	-1,1	1,3	2,0	1,6	1,3	similar to vacuolar protein sorting-associated protein 16 homolog
trans-Golgi/lysosome vesicle transport	Ssc.12623.1.A1_at	***GGA3***	1,2	1,4	1,5	1,8	1,3	1,2	similar to golgi-localized, gamma ear-containing, ARF-binding protein 3
exocytosis	Ssc.11107.1.S1_at	***VAMP3***	1,2	1,3	1,0	-1,4	-1,7	-1,4	similar to vesicle-associated membrane protein 3
endosome/Golgi vesicle transport	Ssc.4135.2.A1_at	***AP1S2***	1,3	1,4	1,4	-1,2	-1,9	1,0	similar to AP-1 complex subunit sigma-2
ER/Golgi vesicle transport	Ssc.28472.1.S1_at	***GOSR2***	1,1	1,1	1,2	-1,4	-1,3	-1,9	similar to golgi SNAP receptor complex member 2
Diverse									
NO regulation	Ssc.29002.1.A1_at	***sGC/GUCY1B3***	1,3	1,2	-1,1	2,2	2,3	2,7	Guanylate cyclase soluble subunit beta-1
NO regulation	Ssc.12191.1.A1_at	***HSP90***	2,4	2,2	1,1	1,8	3,3	4,8	Heat shock protein HSP 90-alpha
NO regulation / ion transport	Ssc.10453.1.S1_at	***CP***	1,4	1,5	1,2	0,9	0,4	0,7	similar to ceruloplasmin
NO regulation	Ssc.21987.1.A1_at	***IFRD1***	1,2	1,4	1,5	-1,5	-1,8	-1,6	Interferon-related developmental regulator 1
